# Voltage-sensor conformational microenvironments encode isoform-selective ion channel activation

**DOI:** 10.1073/pnas.2616367123

**Published:** 2026-09-23

**Authors:** Rían W. Manville, Lorenzo Foglia, Ryan F. Yoshimura, Daniel E. Santos, Alvin Yu, Geoffrey W. Abbott

**Affiliations:** ^a^ Bioelectricity Laboratory, Department of Physiology and Biophysics, School of Medicine, University of California, Irvine, CA 92697; ^b^ Yu Lab, Department of Physiology and Biophysics, School of Medicine, University of California, Irvine, CA 92697

**Keywords:** carnosic acid, Kv7.2, Kv7.3, M-current

## Abstract

Isoform-selective opening of voltage-gated potassium (Kv) channels is desirable for its therapeutic potential yet mechanistically little-understood. Rosemary leaf metabolite carnosic acid (CA) is a high-efficacy opener of the neuronal Kv7.3 channel but exerts minimal effects on Kv7.2 or Kv7.2/3 heteromers. Here, using alanine-scanning mutagenesis, electrophysiology, radioligand binding, and all-atom molecular dynamics simulations, we found that CA achieves high-efficacy activation of Kv7.3 by binding at the extracellular cap of the voltage-sensing domain (VSD), independent of the canonical retigabine pore binding site. Kv7.3 samples a binding-competent VSD conformation absent in Kv7.2, characterized by an expanded extracellular pocket and a favorable electrostatic environment. Central to this mechanism, Kv7.3-L226 functions as a hydrophobic latch that tunes the energetic landscape of the voltage sensor to control ligand efficacy. Unexpectedly, CA potentiates Kv7.2/3 activation by retigabine via ligand-initiated positive allosteric coupling between the VSD and pore, thus enhancing the anticonvulsant action of retigabine despite CA lacking standalone anticonvulsant activity. The findings establish a mechanistic framework in which isoform selectivity arises from VSD conformational microenvironments rather than canonical binding determinants and highlight the voltage sensor as a tunable target for developing selective Kv channel openers.

## Significance

Developing drugs that selectively target closely related ion channel isoforms remains a major challenge because these proteins often share highly conserved drug-binding sites. Here, we show that selective activation of Kv7 potassium channel isoforms by the rosemary-derived metabolite carnosic acid (CA) arises not from unique binding residues, but from subtle conformational and electrostatic differences within the channel voltage sensor. We further show that CA unexpectedly enhances the activity of the anticonvulsant retigabine despite exhibiting minimal activity on its own at therapeutically important Kv7.2/3 channels. These findings establish a general mechanism for isoform-selective ion channel modulation and identify the voltage sensor as a tunable target for developing safer and more selective therapeutics.

Voltage-gated potassium (Kv) channels regulate electrical excitability, excitable cell repolarization, fluid and ion homeostasis, solute transporter function, and hormone production and secretion, among other roles. The Kv7 (*KCNQ*) channel family is comprised of five isoforms (Kv7.1-5) and is highly influential in physiology, with genetic disruption of each of the five members causing human disease ranging from cardiac arrhythmias (Kv7.1), deafness (Kv7.1 and Kv7.4), and developmental and/or epileptic encephalopathies (Kv7.2, Kv7.3, and Kv7.5). Loss of function is the most common form of pathologic dysfunction for Kv channels in general, and for Kv7 channels particularly. Therefore, channel openers (that augment peak current and/or facilitate opening at more hyperpolarized membrane potentials) are sought after for their therapeutic potential.

Among various safety and efficacy considerations, Kv7 isoform specificity is a desirable but uncommon feature of Kv7 channel openers. The first-in-class anticonvulsant retigabine (RTG) activates neuronal Kv7.2-5 channels by binding to a conserved tryptophan in the S5 transmembrane domain (e.g., Kv7.3-W265) (1). RTG also activates heteromeric Kv7.2/3 channels, a key molecular correlate of the neuronal M-current (2, 3). The inhibitory neurotransmitter, GABA, and the related drug gabapentin similarly depend on this tryptophan residue, activating Kv7.3 and Kv7.5 (and Kv7.2/3) but not homomeric Kv7.1, Kv7.2, or Kv7.4 (4, 5). Notably, GABA has been shown to physically bind across Kv7.2-5 (4) isoforms, indicating that binding alone does not determine functional selectivity. These observations highlight a key unresolved question: how isoform-selective activation arises despite shared binding determinants across Kv7 channels.

We previously found that carnosic acid (CA), a benzenediol abietane diterpene produced by *Salvia rosmarinus* (rosemary) and other *Salvia* species (sages), is a highly efficacious opener of Kv7.3, with weaker effects on Kv7.5 and negligible activity at Kv7.2 (6). Unlike RTG, GABA, and gabapentin, CA does not open heteromeric Kv7.2/3 channels, indicating that Kv7.2 confers dominant insensitivity in these assemblies. Consistent with its Kv7.3/5 activity, we previously showed CA counteracts cocaine-induced downregulation of Kv7.3/5 channels in the globus pallidus externus (GPe) and reduces cocaine-seeking behavior in mice (7), highlighting its therapeutic potential. Given this isoform selectivity, along with its brain penetrance (8, 9), and favorable tolerability profile (10), we investigated the molecular basis of CA activity at Kv7.3 and its interactions with Kv7.2/3-targeting modulators.

Here, we identify Kv7.3-specific S4 architecture that defines a VSD conformational microenvironment permissive for CA binding and activation, providing a mechanistic framework for isoform-selective channel modulation. Central to this mechanism, residue L226 functions as a hydrophobic latch that tunes the energetic landscape of the voltage sensor and thereby controls ligand efficacy. Unexpectedly, we found that CA potentiates RTG-mediated activation of Kv7.2/3 channels despite lacking standalone activity, revealing positive allosteric coupling between VSD- and pore-targeted ligands. Consistent with this, coadministration of CA with a subtherapeutic dose of RTG reduces seizure-related mortality in mice. Together, these findings provide a framework for the development of safer, more selective and more effective Kv7-targeted therapeutics.

## Results

### CA Activates Kv7.3 Independently of the Canonical Pore-Binding Site.

**Fig. 1.**
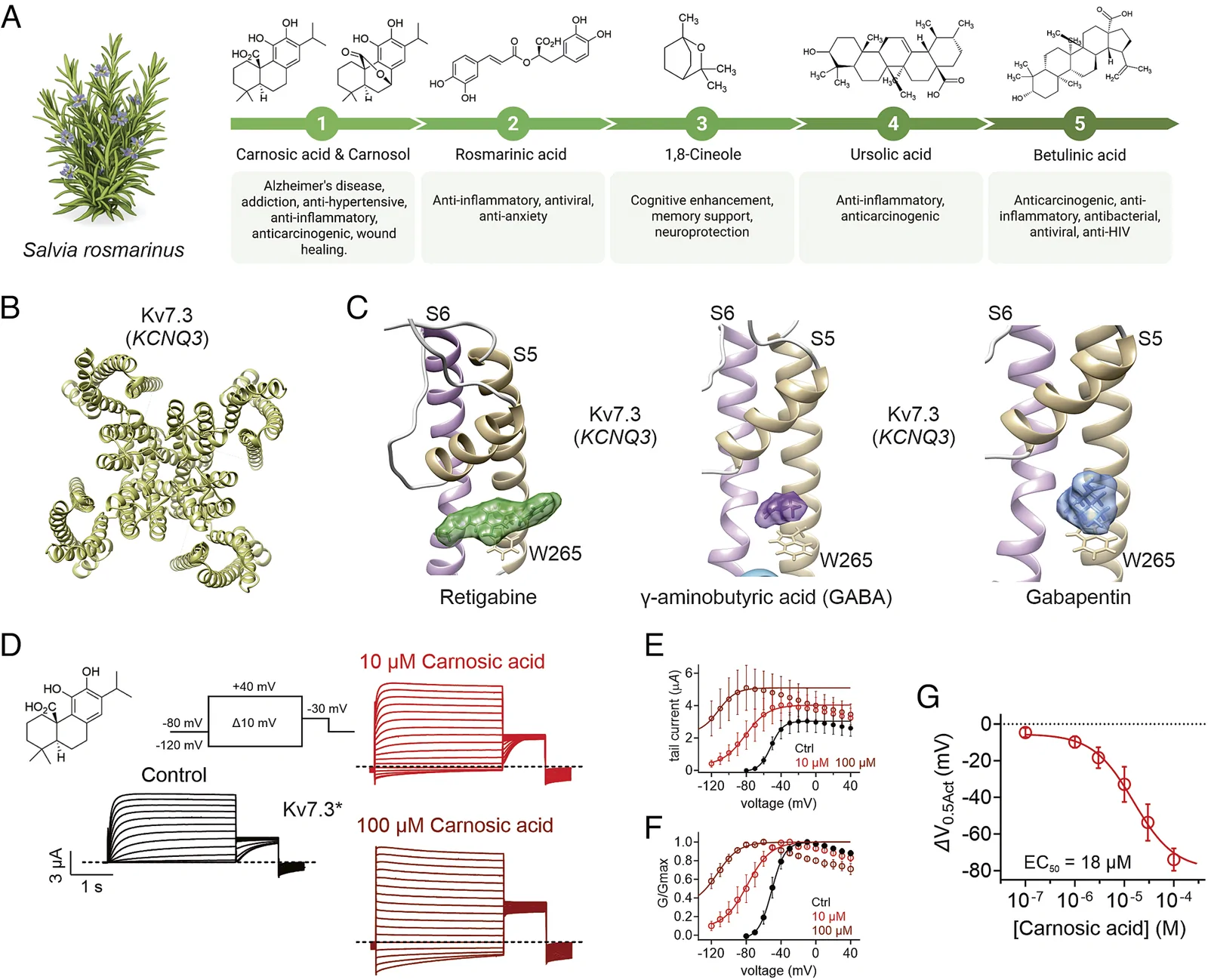
CA from rosemary is an efficacious Kv7.3 opener. Error bars indicate SEM. n denotes biologically independent oocytes. Voltage protocols and dashed zero-current lines are used throughout. (*A*) *S. rosmarinus* (rosemary) and major bioactive constituents. Image generated using Biorender. (*B*) Top–down view of homomeric human Kv7.3. (*C*) Unbiased docking poses of retigabine (green), GABA (purple), and gabapentin (blue) at W265 of the Kv7.3 pore domain. AlphaFold2 model of Kv7.3. (*D*) Mean traces for Kv7.3* channels in the absence (Control; black) or presence of 10 µM (red) and 100 µM (burgundy) CA. Scale bar *Lower Left* for each trace. Chemical structure *Upper Left Inset*; voltage protocol *Upper Inset*; *n* = 5. (*E*) Mean tail current from *D*; *n* = 5. (*F*) G/Gmax from *D*; *n* = 5. (*G*) Mean ΔV_0.5act_ vs. [CA] for oocytes expressing Kv7.3*; *n* = 5.

Evergreen flowering plant *S. rosmarinus* (rosemary) produces various metabolites associated with its purported therapeutic activity, including CA (Fig. 1*A*). Previously, we showed that CA is a highly efficacious, isoform-selective opener of Kv7.3 that acts independently of the canonical RTG binding site that incorporates tryptophan 265 (W265) in S5 (6).

Several structurally diverse ligands—including RTG, but also the inhibitory neurotransmitter γ-aminobutyric acid (GABA), and the anticonvulsant and nerve pain medication gabapentin—require W265 within a canonical small-molecule binding pocket to modulate Kv7.3 activity (1, 4, 5) (Fig. 1 *A*–*C*). Despite sharing this common binding determinant, these ligands exhibit markedly different efficacies, indicating that engagement of the canonical W265 site alone is insufficient to explain the magnitude of Kv7.3 activation. Consistent with this, CA robustly activated Kv7.3*, shifting V_0.5act_ by −33 mV and −74 mV at 10 µM and 100 µM, respectively, with an EC_50_ of 18 ± 5.7 µM (Fig. 1 *D*–*G* and *SI Appendix*, Fig. S1*A* and Table S1). Thus, despite not requiring W265, CA demonstrates that efficacious opening of Kv7.3 can occur through an alternative, noncanonical mechanism.

### The Kv7.3 S4 Architecture Endows Its Higher Sensitivity to CA.

CA strongly hyperpolarizes the V_0.5act_ of Kv7.3* yet does not require W265, indicating that CA acts through a distinct gating-modulatory mechanism. Sequence alignment of Kv7.1–Kv7.5 VSD segments revealed substantial conservation across isoforms but notable divergence within the S4 helix, the principal voltage-sensing element of the channel (*SI Appendix,* Fig. S2*A*). We therefore tested whether the S4 contributes to CA sensitivity by performing alanine-scanning mutagenesis across the entire Kv7.3* S4 segment.

**Fig. 2.**
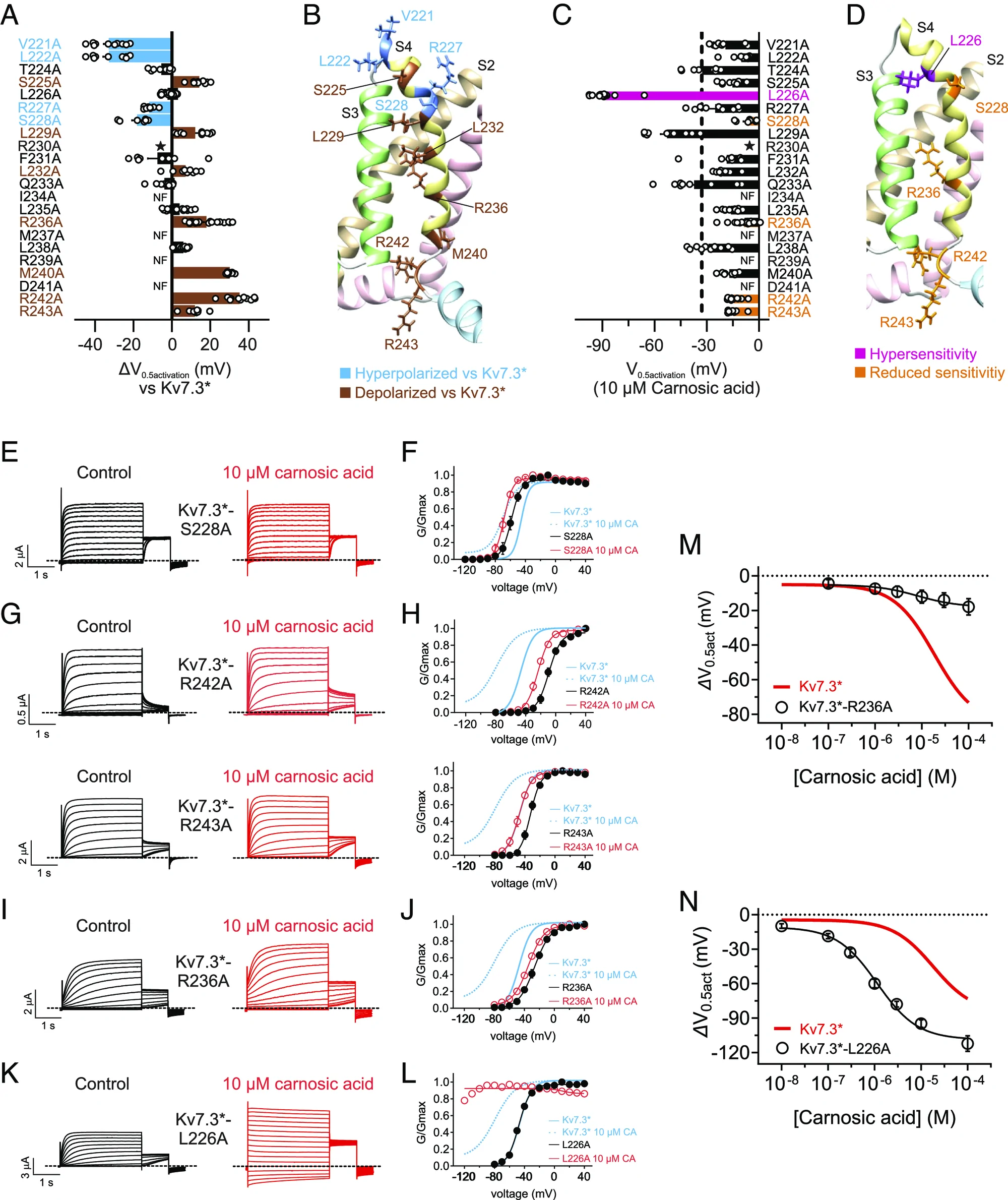
Alanine scanning of Kv7.3 S4 identifies its extracellular face as the determinant of CA hypersensitivity. (*A*) Baseline V_0.5act_ of Kv7.3* S4 alanine mutants; *n* = 7 to 18. Black star, Constitutively active, V_0.5act_ not possible to quantify. NF, nonfunctional. (*B*) AlphaFold2 model of Kv7.3 VSD highlights residues causing hyperpolarizing (blue) or depolarizing (brown) shifts in V_0.5act_. (*C*) CA (10 µM) responses of Kv7.3* S4 alanine mutants. The dashed line represents Kv7.3*; *n* = 7 to 18. Black star, Constitutively active channel, V_0.5act_ not possible to quantify. NF, nonfunctional. (*D*) AlphaFold2 model of Kv7.3 showing the VSD with residues conferring CA hypersensitivity (magenta) or reduced sensitivity (orange). (*E*) Mean traces for Kv7.3*-S228A in the absence (Control; black) or presence of CA (10 µM) (*red*); *n* = 7. Scale bars *Lower Left*. (*F*) G/Gmax from *E*; *n* = 7. Kv7.3* fits shown in cyan. (*G*) Mean traces for Kv7.3*-R242A (*n* = 11) and R243A (*n* = 9) in the absence (Control; *black*) or presence of CA (10 µM; *red*). Scale bars *Lower Left*. (*H*) G/Gmax from G; *n* = 9 to 11. Kv7.3* fits shown in cyan. (*I*) Mean traces for Kv7.3* mutant R236A in the absence (Control; *black*) or presence of CA (10 µM) (*red*); *n* = 18. Scale bars *Lower Left*. (*J*) G/Gmax from *I*; *n* = 18. Kv7.3* fits shown in cyan. (*K*) Mean traces for Kv7.3*-L226A in the absence (Control; *black*) or presence of CA (10 µM) (*red*); *n* = 7. Scale bars *Lower Left*. (*L*) G/Gmax from K; *n* = 7. Kv7.3* fits shown in cyan. (*M*) Mean ΔV_0.5act_ vs. [CA] for oocytes expressing Kv7.3* (*solid red*) or Kv7.3*-R236A (black); *n* = 6. (*N*) Mean ΔV_0.5act_ vs. [CA] for oocytes expressing Kv7.3* (*solid red*) or Kv7.3*-L226A (black); *n* = 7.

First, we assessed the baseline function of the S4 mutants by comparing the V_0.5act_ to Kv7.3*. Four residues in the extracellular-proximal half of S4 induced a hyperpolarizing shift in the V_0.5act_, with V221A and L22A at the top of the S4 cap shifting the V_0.5act_ −33.3 ± 2.69 and −33.75 ± 2.99, respectively (Fig. 2 *A* and *B* and *SI Appendix*, Fig. S2 *B*–*D* and Table S2). Several mutants located throughout the S4 induced positive shifts in the V_0.5act_, in particular, L232A, R236A, M240A, R242A, and R243A positively shifted the V_0.5act_ between 12 to 30 mV (Fig. 2 *A* and *B* and *SI Appendix*, Fig. S2 *E*–*G* and Table S2). Substitution of R230 in Kv7.3* resulted in constitutively active currents. This same constitutive activation has also been reported for mutations to arginine residues at the reciprocal position in Kv7.1 (R231), Kv7.2 (R201), and Kv7.3 (R230C/H/S) (11–13) (Fig. 2*A* and *SI Appendix*, Fig. S2 *E*–*G* and Table S2).

Substitutions that decreased CA sensitivity were spread along the entire length of the S4 (Fig. 2 *C* and *D*). Alanine substitution of S228 near the S4 cap significantly reduced CA activity, with 10 µM shifting the V_0.5act_ −10 mV (Fig. 2 *E* and *F* and *SI Appendix*, Table S3). Despite being located at the foot of the S4, neighboring residues R242 and R243 also significantly blunted the effect of 10 µM CA, only shifting the V_0.5act_ by −15 mV (Fig. 2 *G* and *H* and *SI Appendix*, Table S3). However, the most substantial reduction in CA sensitivity was observed at R236A, with only an −8 mV shift observed at 10 µM (Fig. 2 *I* and *J* and *SI Appendix*, Table S3). Conversely, L226A, located at the top of the S4 segment, hypersensitized Kv7.3* to CA (Fig. 2 *K* and *L* and *SI Appendix*, Table S3). At 10 µM, CA induced >80% constitutive activation at −120 mV and a striking −89 mV shift in V_0.5act_ (Fig. 2 *C*, *K*, and *L* and *SI Appendix*, Table S3). Next, we investigated the effect of R236A and L226A on overall CA efficacy and potency by conducting dose–response studies.

The R236A mutant markedly reduced CA efficacy, with a maximal shift of −18 ± 4.7 mV observed at 100 µM (Fig. 2*M* and *SI Appendix*, Fig. S3*A* and Table S4). In contrast, the hypersensitive L226A mutant substantially enhanced CA response, producing a −112 ± 6.8 mV shift in V_0.5act_ at 100 µM (Fig. 2*N* and *SI Appendix,* Fig. S3 *B*; *SI Appendix,* *Table S5*). Concurrently, L226A also enhanced CA potency, reducing the EC_50_ to 1.1 µM — an ~16-fold increase in potency relative to Kv7.3*. Alanine-scanning identifies the Kv7.3 S4 as a key determinant of voltage dependence and CA sensitivity. Consistent with this, Kv7.3 diverges from other Kv7 isoforms in this region, with L222 replacing a conserved phenylalanine and lacking an adjacent alanine found near the hypersensitivity residue L226.

### L226 Defines a Hydrophobic Latch That Governs CA Sensitivity.

**Fig. 3.**
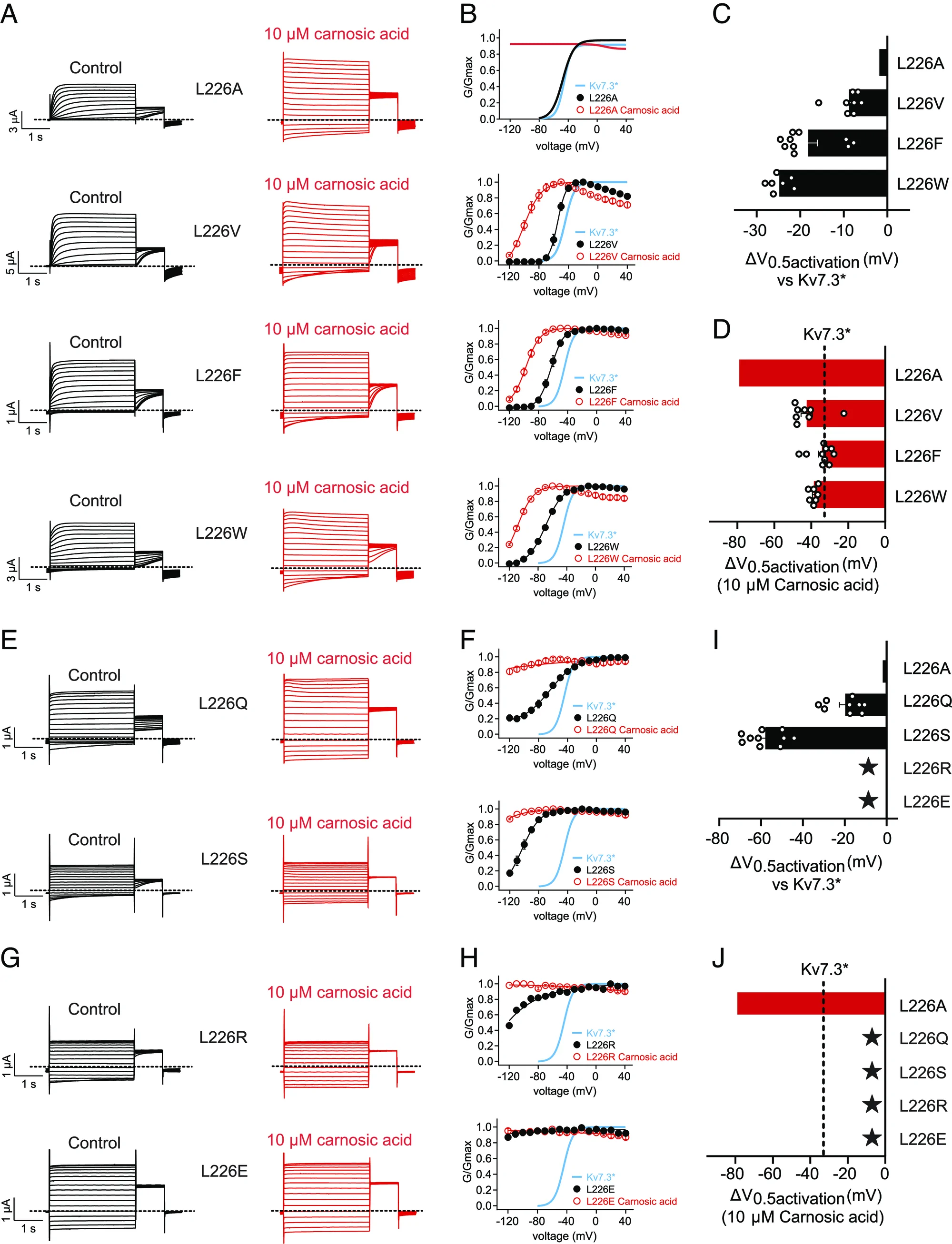
Hydrophobic scan of L226 at the S4 cap reveals hydrophobic packing tunes CA efficacy. (*A*) Mean traces for Kv7.3*-L226A, L226V, L226F, and L226W in the absence (Control; black) or presence of CA (10 µM) (*red*); *n* = 7 to 10. Scale bars Lower *Left*. (*B*) G/Gmax from A; *n* = 7 to 10. Baseline Kv7.3* fits shown in cyan. (*C*) Baseline V_0.5act_ of L226 hydrophobic mutants; *n* = 7 to 10. (*D*) CA responses of L226 hydrophobic mutants; *n* = 7 to 10. (*E*) Mean traces for Kv7.3*-L226Q and L226S in the absence (Control; *black*) or presence of CA (10 µM) (*red*); *n* = 9 to 10. Scale bars *Lower Left*. (*F*) G/Gmax from *E*; *n* = 9 to 10. Baseline Kv7.3* fits shown in cyan. (*G*) Mean traces for Kv7.3*-L226R and L226E in the absence (Control; *black*) or presence of CA (10 µM) (*red*); *n* = 6 to 10. Scale bars *Lower Left*. (*H*) G/Gmax from G; *n* = 6 to 10. Baseline Kv7.3* fits shown in cyan. (*I*) Baseline V_0.5act_ of L226 polar and charged mutants; *n* = 6 to 10. (*J*) CA responses of L226 polar and charged mutants; *n* = 6 to 10.

Having identified L226 as a key determinant of CA sensitivity, we systematically varied the physicochemical properties at this position to define their impact on CA efficacy. Substitution with a small hydrophobic alanine (L226A) markedly enhanced the efficacy of 10 µM CA without altering the baseline V_0.5act_ (Fig. 3 *A*–*D* and *SI Appendix*, Table S5). Replacement with valine (L226V) similarly had minimal effect on baseline gating but did not confer the same hypersensitivity, with 10 µM CA producing a shift (−42.5 ± 3.1 mV) comparable to Kv7.3* (Fig. 3 *A*–*D* and *SI Appendix*, Table S6). In contrast, substitution with bulkier hydrophobic residues, phenylalanine (L226F) and tryptophan (L226W), progressively left-shifted the baseline V_0.5act_ (−17.9 ± 2.2 and −24.8 ± 0.9 mV, respectively) (Fig. 3 *A*−*C* and *SI Appendix*, Tables S7 and S8), indicating increased stabilization of activated states. Despite these baseline shifts, CA efficacy was not enhanced, with both mutants exhibiting responses similar to Kv7.3* (−34.2 ± 1.9 and −38.6 ± 0.8 mV, respectively) (Fig. 3 *A*, *B*, and *D* and *SI Appendix*, Tables S7 and S8).

We next examined the impact of polarity by substituting L226 with glutamine (L226Q) and serine (L226S). Introduction of polar residues markedly destabilized the resting state, shifting the baseline V_0.5act_ by −19.8 ± 2.8 mV (L226Q) and −57.9 ± 2.8 mV (L226S) (Fig. 3 *E*, *F*, and *I* and *SI Appendix,* Tables S9 and S10). Under these conditions, 10 µM CA effectively locked both channels in an activated state, producing ~80% activation at −120 mV (Fig. 3 *E*, *F*, and *J*). Finally, substitution with charged residues, arginine (L226R) and glutamic acid (L226E), abolished the resting state altogether, resulting in constitutive activation at baseline (Fig. 3 *G*–*I*). Consequently, the effect of 10 µM CA was obscured, as both mutants already exhibited substantial activation at −120 mV (Fig. 3 *G*, *H*, and *J*).

Together, these results are consistent with L226 functioning as a hydrophobic latch-like element that stabilizes the S4 segment in its resting configuration. Mutations that weaken this latch enhance CA-mediated activation, whereas substitutions that strengthen or disrupt it reduce or obscure CA efficacy. Thus, CA sensitivity is governed not by binding alone, but by the available energetic “headroom” for S4 activation.

### Binding Mechanism and Affinity of CA for Kv7.3 and Kv7.2.

Microsecond-timescale all-atom molecular dynamics (MD) can elucidate the binding pathways, the atomic interactions that promote and stabilize binding, and the conformational changes that enable protein function (14–22). In this study, we use all-atom MD to reveal the molecular mechanisms by which CA activates Kv7.3 in a subtype selective manner (4, 23–26) (*SI Appendix*, Fig. S4).

**Fig. 4.**
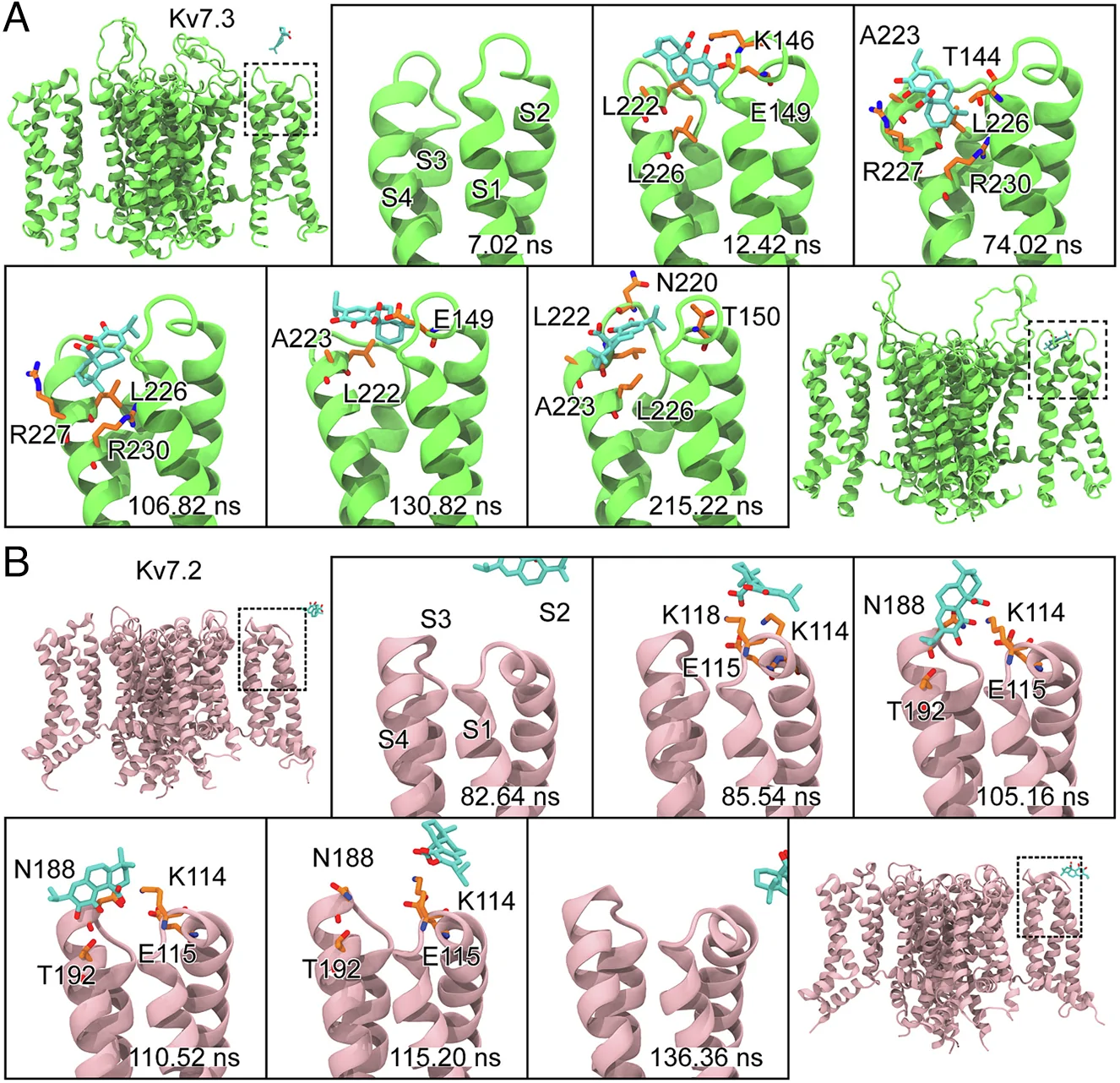
Molecular mechanism of CA binding to Kv7.3 and Kv7.2. Time-resolved MD trajectories showing spontaneous CA association with Kv7.3 (*A*) and Kv7.2 (*B*). *Insets* show enlarged views of the extracellular VSD. In Kv7.3, CA progresses into site 3 where it is stabilized by K146, E149, N220, L222, A223, L226, and T150. In Kv7.2, CA forms only transient interactions before dissociating into bulk solvent.

In aggregate, we collected 1.5 microseconds of atomistic MD sampling for Kv7.3 across five trajectories. In all trajectories, CA spontaneously associates with an extracellular pocket of the VSD, and in three of the five trajectories, interactions between CA and Kv7.3 led to stable binding. CA initially forms electrostatic contact with K146 (Fig. 4*A* and Movie S1). This electrostatic interaction pulls the ligand to the interior of the VSD, where it engages E149 and is stabilized by L222 and L226 (Movie S2). At 74.02 ns, the hydroxyl groups of CA form hydrogen bonds with R227 and R230, and the ligand inserts more deeply into the VSD. CA interactions with R227 and R230 weaken and break at 106.82 ns and 130.82 ns, during which the ligand rotates and settles into a stable binding pose supported by hydrophobic interactions with L222, A223, L226, and T150, and contact with the peptide backbone of N220 at 215.22 ns.

Notably, the L226 sidechain frequently rotates between two orientations (Movie S2): one in which a single methyl group contacts the methyl group of CA (Fig. 4*A*, 106.82 ns), and one in which both methyl groups form a bidentate hydrophobic contact with the CA methyl group (Fig. 4*A*, 215.22 ns). The latter conformation inserts CA deeper within the VSD cavity, consistent with a hydrophobic latch that stabilizes the CA bound pose (*SI Appendix,* Fig. S5). In agreement with this mechanism, our mutagenesis experiments show that substituting L226 with other hydrophobic residues preserves CA efficacy. In particular, the L226A mutant markedly enhances CA efficacy, likely because the smaller alanine side chain frees space that allows CA to adopt a more favorable binding pose deeper in the VSD cavity.

Mutagenesis of S228 and R236 also reduced CA efficacy, although neither residue contacts CA in the stably bound pose. Analysis of S228 during the MD simulations indicate that S228 modulates the side chain orientations of the neighboring R227 and R230 residues and is involved in maintaining the binding pocket residues L226, R227, S228, L229, and R230 in a CA-binding competent conformation (*SI Appendix,* Fig. S6 *A–C*). Measurement of the dihedral angle formed by the Cα atoms of L227, R227, S228, and L229 ($\varphi_{1}$) as well as the Cα atoms of R227, S228, L229, and R230 ($\varphi_{2}$) of Kv7.3 across the CA-bound, and apo, closed, and open states revealed that the microenvironment of the CA-bound conformation is more similar to that of the open state (*SI Appendix,* Fig. S6*D*). Additionally, three of these neighboring residues (L226, R227, and R230) form direct contacts with CA during the simulation. Consequently, mutations at S228 may alter the side-chain orientations of these adjacent residues, thereby reducing the affinity between CA and the extracellular binding pocket in the VSD.

R236 on the other hand, is located near the C-terminal of the S4 helix and forms a stable salt bridge interaction with E160 on the S2 helix (*SI Appendix,* Fig. S7*A*). Analysis of the correlated motions via dynamic cross-correlation calculations of trajectories of the CA-bound state, and the apo, closed, and open states (*SI Appendix,* Fig. S7 *B* and *C*) revealed that the S2 and S4 helices are positively correlated. Mutations to the salt bridge, for instance R236A, would be expected to disrupt S2–S4 coupling and propagation of CA-induced VSD conformational changes to the pore via the S4 and the S4–S5 linker. Similar disruptions to the salt-bridge interaction and thus VSD-pore coupling, have been previously measured in electrophysiology recordings of a R236C variant of Kv7.3 (27).

Despite the homology between Kv7.2 and Kv7.3, CA showed markedly reduced stability at the extracellular VSD site in Kv7.2. In MD simulations of Kv7.2, the ligand repeatedly associated with and dissociated from an extracellular binding site analogous to that found in Kv7.3 (Movie S3), consistent with its lower binding affinity for Kv7.2. Initial ligand contact occurs through K114 and K118, which transiently pull the ligand toward the interior of the VSD (Fig. 4*B*). However, unlike with Kv7.3, the ligand does not diffuse deeper into the VSD or adopt a stable binding pose. Instead, the local chemical moiety of the VSD in Kv7.2 does not support favorable ligand orientation, and unfavorable interactions with E115, N188, and T192 promote ligand destabilization and dissociation back into bulk solvent after 136 ns (Movie S4).

### MD Simulations of Kv7.3 Reveal Preferred Binding Sites for CA.

**Fig. 5.**
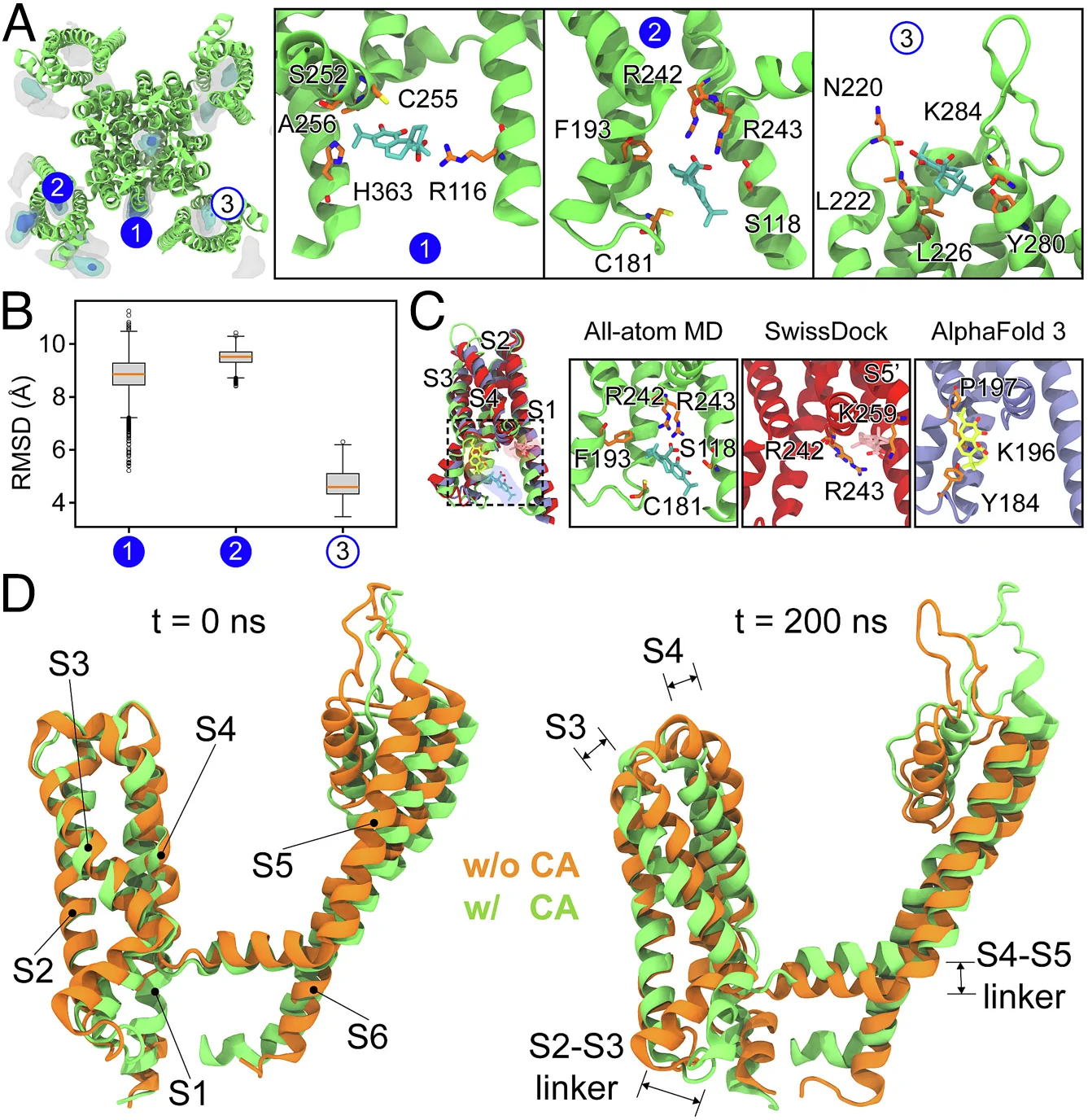
Kv7.3 binding sites for CA and conformational change. (*A*) Ligand density map highlighting the spatial positions of CA binding. Contours indicate 50% (dark blue), 25% (cyan), and 5% (gray) of the probability density. Binding sites are indicated as filled (intracellular) or open (extracellular) circles with magnified views in the *Right* panels. (*B*) Ligand stability is evaluated at each of the three sites using RMSD. The median RMSD is shown as an orange line, and the 1st and 3rd quartiles are boxed. Bars represent a 1.5× the interquartile range, and outliers are shown as circles. (*C*) Comparison of binding site 2 detected from all-atom MD, SwissDock, and AlphaFold2. (*D*) Alignment of a Kv7.3 monomer at the initial and final timesteps of MD simulations in the absence (orange) and presence (green) of CA. Conformational changes in Kv7.3 induced by CA binding to binding site 3 are indicated with arrows.

Across the MD trajectories, CA transiently bound to multiple regions in Kv7.3. To identify potential ligand-binding sites, we computed a three-dimensional ligand density map from the all-atom MD simulation data (26) (Fig. 5*A*). Analysis of the probability density, ρ(r), revealed three high-occupancy sites, defined as contiguous volumes exceeding 50% of the maximum density. These sites were localized along the S1–S4 helices of the VSD and at the S4–S5 linker, which mechanically couples the VSD to the pore domain (28, 29).

At site 1, R116 and C255 form stable hydrogen bonds with the hydroxyls of the ligand, while H363, A256, and S252 stabilize its methyl and the aromatic groups through nonpolar interactions. This intersubunit site, located between helices S1 and S6 is close to the homologous binding site of Lu AG00563 on Kv3.1 (30). In site 2, the arginine pair R242/R243 anchors CA’s carboxyl group via hydrogen bonds, and F193 stabilizes its aromatic ring. This intracellular VSD site matches the reported binding site of ztz-240 on Kv7.2 (31). In site 3, K284 and N220 form hydrogen bonds with the ligand’s hydroxyls, while L222, L226, and Y280 provide hydrophobic interactions with the nonpolar groups of the ligand, consistent with the NH29 binding pocket on Kv7.2 (32). Therefore, the binding sites identified from the density analysis agree with known interactions at Kv channels, namely those of Lu AG00563, ztz-240, and NH29.

To compare the relative stability of CA at the identified sites, we performed MD simulations with the ligand simultaneously bound at all three locations (Movie S5) and quantified positional stability of the ligand using RMSD measurements calculated relative to the initial binding pose (Fig. 5*B*). Sites 1 and 2 showed comparable stability, with site 1 exhibiting a lower median but greater variability compared to site 2. Site 3, an extracellular pocket of the VSD that is readily accessible from bulk solvent, showed the lowest RMSD, indicating the most stable ligand pose. Site 3 is lined by L222 and L226, residues at which substitution alters CA sensitivity (Fig. 2). Sites 1 and 2 likely correspond to auxiliary sites as we have identified in other protein–ligand systems, which represent metastable ligand interaction sites or pockets at which other molecules bind (26).

In addition, we compared ligand binding sites from our MD simulations with poses derived from molecular docking [SwissDock (33)] and structure prediction [AlphaFold (34)]. Both methods docked CA to the VSD at sites that showed a high similarity and proximity to site 2 derived from our MD trajectories (Fig. 5*C*). Interestingly, SwissDock finds stable contacts between CA and three residues adjacent to the S4–S5 linker: K259, R242, and R243. The binding site from AlphaFold is also at the base of the VSD, although the ligand forms stable contacts with Y184, K196, and P197.

To assess the conformational changes in Kv7.3 that occur in response to CA binding, we compared trajectories in the presence and absence of CA after 200 ns of simulated time (Fig. 5*D*). When CA occupies the extracellular cavity of the VSD, it induces substantial bending of the S3 and S4 helices, shifting them by 34° and 33°, respectively, along their principal axis. These changes propagate as a 6.9 Å shift in the S4–S5 linker that lifts the pore helices in a manner consistent with altered VSD-pore coupling (35, 36). This mechanism centers on residues R242 and R243, which function as a pivot that anchors the amino-terminal end of the S4–S5 linker to motions of the S2, S3, and S4 helices.

Given that the CA binding site is situated in the VSD, we next asked whether CA activation of Kv7.3* is state dependent. We examined Kv7.3* activation following CA application under different voltage-state conditions, as previously described with ICA73 and retigabine (37). When CA was applied while channels were held at strongly hyperpolarized potentials (−120 mV), the first depolarizing pulse and subsequent pulses elicited little instantaneous current (*SI Appendix,* Fig. S8 *A* and *B*). However, when holding Kv7.3* at −80 and −60 mV, a larger standing current was observed at the first pulse and became progressively more pronounced with subsequent pulses, consistent with voltage-sensor activation facilitating CA efficacy (*SI Appendix,* Fig. S8 *C–F*). MD simulations showed the binding pocket for CA in the VSD was more accessible in the open than closed state (*SI Appendix,* Fig. S9 *A* and *B*), and RMSD of the VSD relative to the CA-bound state was lower in the open state (<6 Å) than closed state (>8 Å) throughout the simulation (*SI Appendix,* Fig. S9*C*). Together, these findings indicate CA activation of Kv7.3* is state-dependent and is enhanced in the open state.

### S4 Cap Divergence Underlies CA Selectivity for Kv7.3 over Kv7.2.

**Fig. 6.**
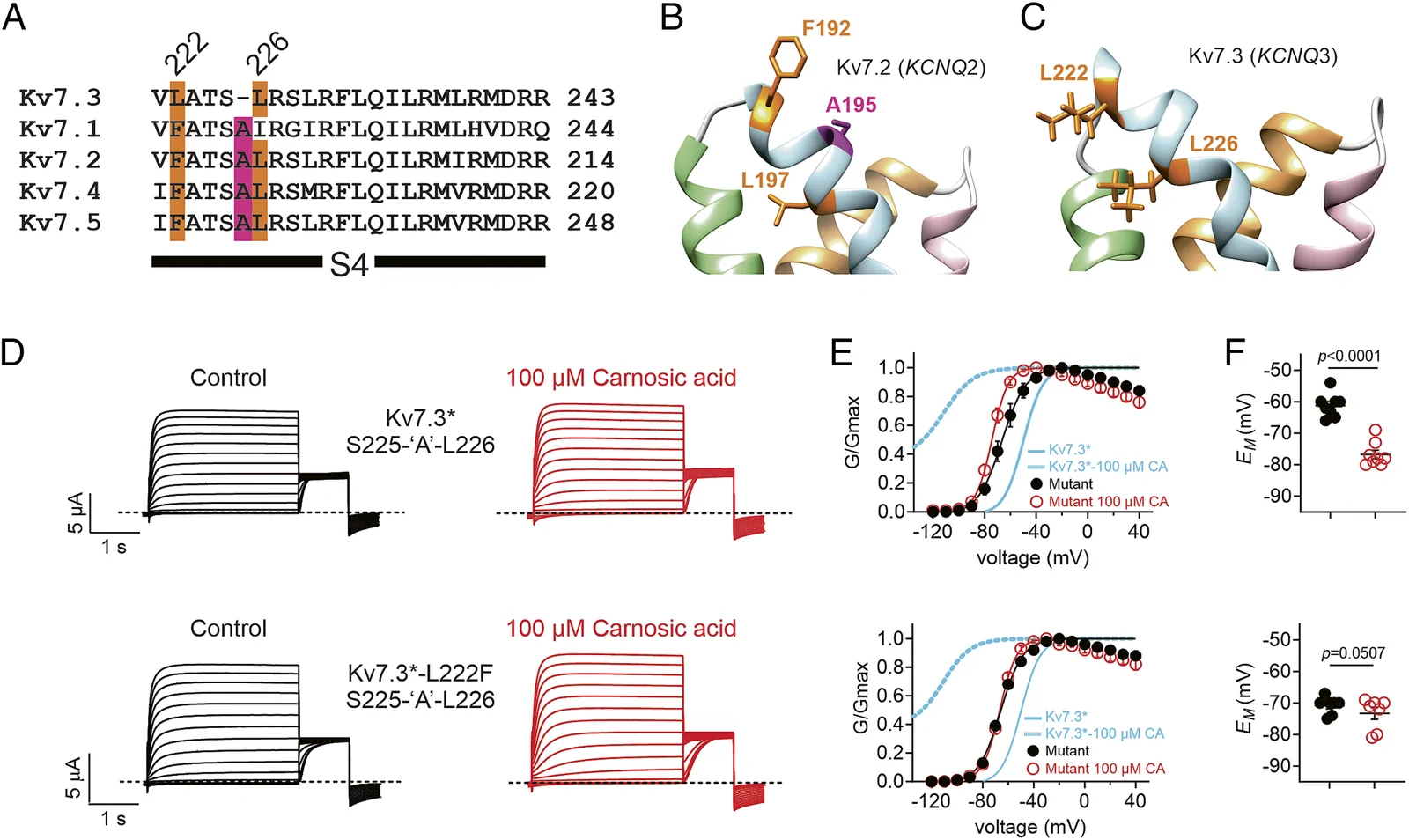
Kv7.3 S4 sequence divergence underlies its high sensitivity to CA compared to Kv7.2 (*A*) Sequence alignment of human Kv7 S4 segments with notable divergent residues highlighted. (*B*) Cryo-EM structure of Kv7.2 (PDB: 7CR0) (31) showing the extracellular-facing half of the VSD, with residues highlighted as in panel *A*. (*C*) AlphaFold2 model of Kv7.3 showing the extracellular-proximal half of the VSD, with residues highlighted as in panel *A*. (*D*) Mean traces for (*Upper*) Kv7.3*-S225-A-L226 and Kv7.3*-L222F,S225-A-L226 homomeric channels in the absence (Control; *black*) or presence of CA (100 µM) (*red*); *n* = 7 to 8. Scale bars *Lower Left*. (*E*) G/Gmax from *D*; *n* = 7 to 8. Baseline Kv7.3* fits shown in cyan. (*F*) Resting membrane potential from *D*; *n* = 7 to 8.

The alanine-scanning mutagenesis (Fig. 2) and MD simulations (Figs. 4 and 5) implicate the extracellular S4 cap of Kv7.3 as a potential determinant of both CA activation and isoform selectivity. Specifically, a leucine at position 222 (phenylalanine in all the other Kv7 channels) and the absence of an adjacent alanine residue present in all other Kv7 isoforms (Fig. 6 *A*–*C*). Therefore, we modified the Kv7.3 S4 cap to resemble the other Kv7 channel isoforms.

We first inserted the missing alanine between S225 and L226 of Kv7.3 (Kv7.3*-S225–A–L226), corresponding to the position conserved in the other Kv7 isoforms. Strikingly, this insertion almost completely blunted CA sensitivity, with Kv7.3*-S225-‘A’-L226 exhibiting only a small hyperpolarizing shift in V_0.5act_ (−8.0 ± 2.7 mV at 100 µM) compared with the robust shift observed in Kv7.3* (−76 mV at 100 µM) (Fig. 6 *D*–*F*, *Top* and *SI Appendix*, Table S11). CA was still able to significantly shift the *E_M_* of unclamped oocytes expressing Kv7.3*-S225-‘A’-L226 (Fig. 6 *F*, *Top*). Next, we substituted leucine 222 with phenylalanine (L222F) in the Kv7.3*-S225–A–L226 background to generate the Kv7.3*-L222F-S225–A–L226 construct, thereby fully recapitulating the S4 cap architecture found in the other Kv7 isoforms. Kv7.3*-L222F-S225–A–L226 completely abolished Kv7.3* CA sensitivity, shifting V_0.5act_ by −0.78 ± 0.28 mV at 100 µM (Fig. 6 *D*–*F*, *Bottom* and *SI Appendix*, Table S12). Concurrently, 100 µM CA had no effect on the *E_M_* of unclamped oocytes expressing Kv7.3*-L222F-S225–A–L226 (Fig. 6 *F*, *Bottom*).

**Fig. 7.**
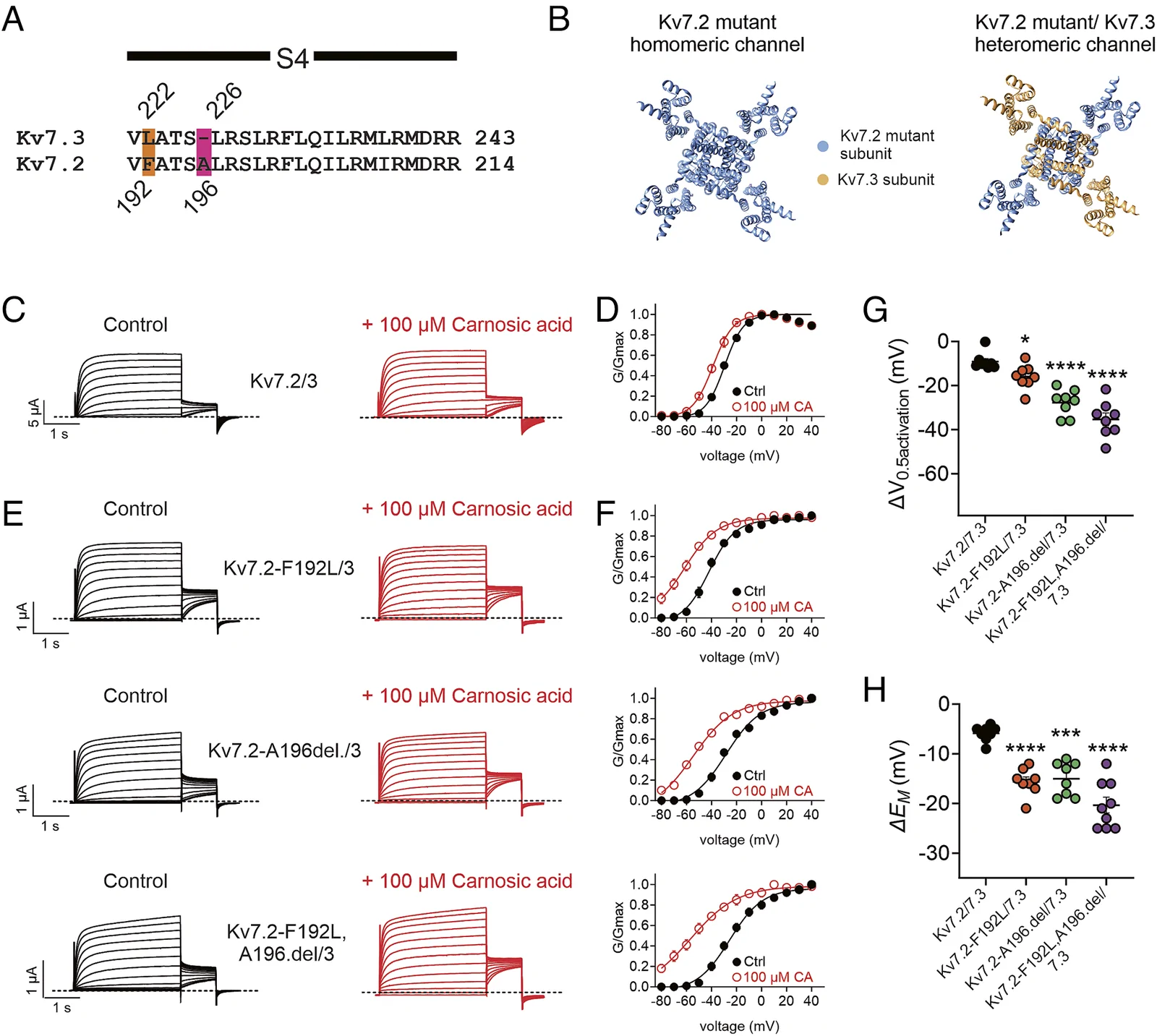
Kv7.3-like S4 substitutions in Kv7.2 endow Kv7.2/3 with CA sensitivity. (*A*) Sequence alignment of Kv7.3 and Kv7.2 S4 segments with notable divergent residues highlighted. (*B*) Subunit composition of Kv7.2 mutant/Kv7.3 heteromer combinations injected. (*C*) Mean traces for Kv7.2/3 heteromers in the absence (Control; black) or presence of 100 µM CA (*red*). Scale bars *Lower Left*; *n* = 8. (*D*) G/Gmax from *C*; *n* = 8. (*E*) Mean traces for Kv7.2-F192L/7.3, Kv7.2-A196del/7.3, and Kv7.2-F192L, A196del/7.3 heteromeric channels in the absence (Control; *black*) or presence of 100 µM CA (*red*); *n* = 8. Scale bars *Lower Left*. (*F*) G/Gmax from *E*; *n* = 8. (*G*) Summary of V_0.5act_ shifts in response to 100 μM CA, from *C* to *F*; *n* = 8. *P = 0.0375, ****P < 0.0001. One-way ANOVA with Dunnett’s correction. (*H*) Summary of *E_M_* shifts in response to 100 μM CA, from *C* to *F*; *n* = 8. ***P = 0.0001, ****P < 0.0001. One-way ANOVA with Dunnett’s correction.

These findings provide a compelling molecular basis for the selectivity of CA for Kv7.3 over other Kv7 isoforms. To further test this hypothesis, we performed the reciprocal experiment in Kv7.2, the principal heteromeric partner of Kv7.3 in the neuronal M-current. Specifically, we introduced the Kv7.3-specific S4 cap features into Kv7.2 by substituting the phenylalanine corresponding to Kv7.3-L222 (Kv7.2-F192L) and deleting the adjacent alanine preceding the Kv7.3-L226 equivalent (Kv7.2-A196del) to determine whether these changes could confer CA sensitivity to Kv7.2, which is otherwise insensitive (Fig. 7*A*). Modifications to the S4 cap of Kv7.2 rendered the homomeric Kv7.2-F192L, Kv7.2-A196del, and Kv7.2-F192L, A196del channels nonfunctional (*SI Appendix*, Fig. S10 *A* and *B* and Table S13). As Kv7.2 is the principal heteromeric partner of Kv7.3 in the neuronal M-current we leveraged this and tested CA sensitivity of Kv7.3/Kv7.2-F192L, Kv7.2-A196del (and single-mutant) heteromers (Fig. 7*B*). Heteromeric channels formed by Kv7.2-F192L/7.3, Kv7.2- A196del/7.3, and Kv7.2-F192L, A196del /7.3 were all functional. Although the peak current magnitudes were approximately one-tenth those of wild-type Kv7.2/3 heteromers, they remained substantially larger than currents recorded from Kv7.3 homomeric channels (*SI Appendix*, Fig. S10 *C* and *D* and Table S14). Examination of the baseline V_0.5act_ revealed only a mild left- or right-shift compared to wild-type Kv7.2/3 (*SI Appendix*, Fig. S10*E* and Table S15). To further confirm incorporation of the mutant Kv7.2 subunits into heteromeric channels, we examined the effects of the Kv7.2-selective openers ICA06973, ICA27243, and ICA110381 (38–40). At 10 µM, ICA06973, ICA27243, and ICA110381 shifted the V_0.5act_ of wild-type Kv7.2/3 channels by −13, −17, and −26 mV, respectively (*SI Appendix,* Fig. S11 *A–D*). Similarly, Kv7.2-F192L/Kv7.3, Kv7.2-A196del/Kv7.3, and Kv7.2-F192L,A196del/Kv7.3 heteromers exhibited comparable sensitivity to all three Kv7.2-selective openers (*SI Appendix,* Fig. S12 *A–I*), confirming functional incorporation of the mutant Kv7.2 subunits into the heteromeric channels.

As reported previously, Kv7.2/3 channels were only mildly sensitive to 100 µM CA, shifting V_0.5act_ by −9.1 ± 1.3 mV (Fig. 7 *C* and *D* and *SI Appendix*, Table S16). By comparison, Kv7.2-F192L/7.3 and Kv7.2-A196del/7.3 heteromers exhibited increased sensitivity, shifting V_0.5act_ by −20.3 ± 3.9 mV and −24.6 ± 1.1 mV, respectively (Fig. 7 *E*–*G* and *SI Appendix*, Tables S17 and S18). The double-mutant heteromer Kv7.2-F192L, A196del/7.3 exhibited the greatest increase in CA sensitivity, shifting V_0.5act_ by −36.3 ± 4.1 mV (Fig. 7 *E*–*G* and *SI Appendix*, Table S19). All mutant heteromer combinations shifted the *E_M_* of unclamped oocytes (Fig. 7*H*). Collectively, these findings demonstrate that the Kv7.3-selective action of CA is determined by the two isoform-specific sequence differences within the S4 cap.

### Mutations to the Extracellular S3vS4 Loop Remodel Site 3 in the VSD.

**Fig. 8.**
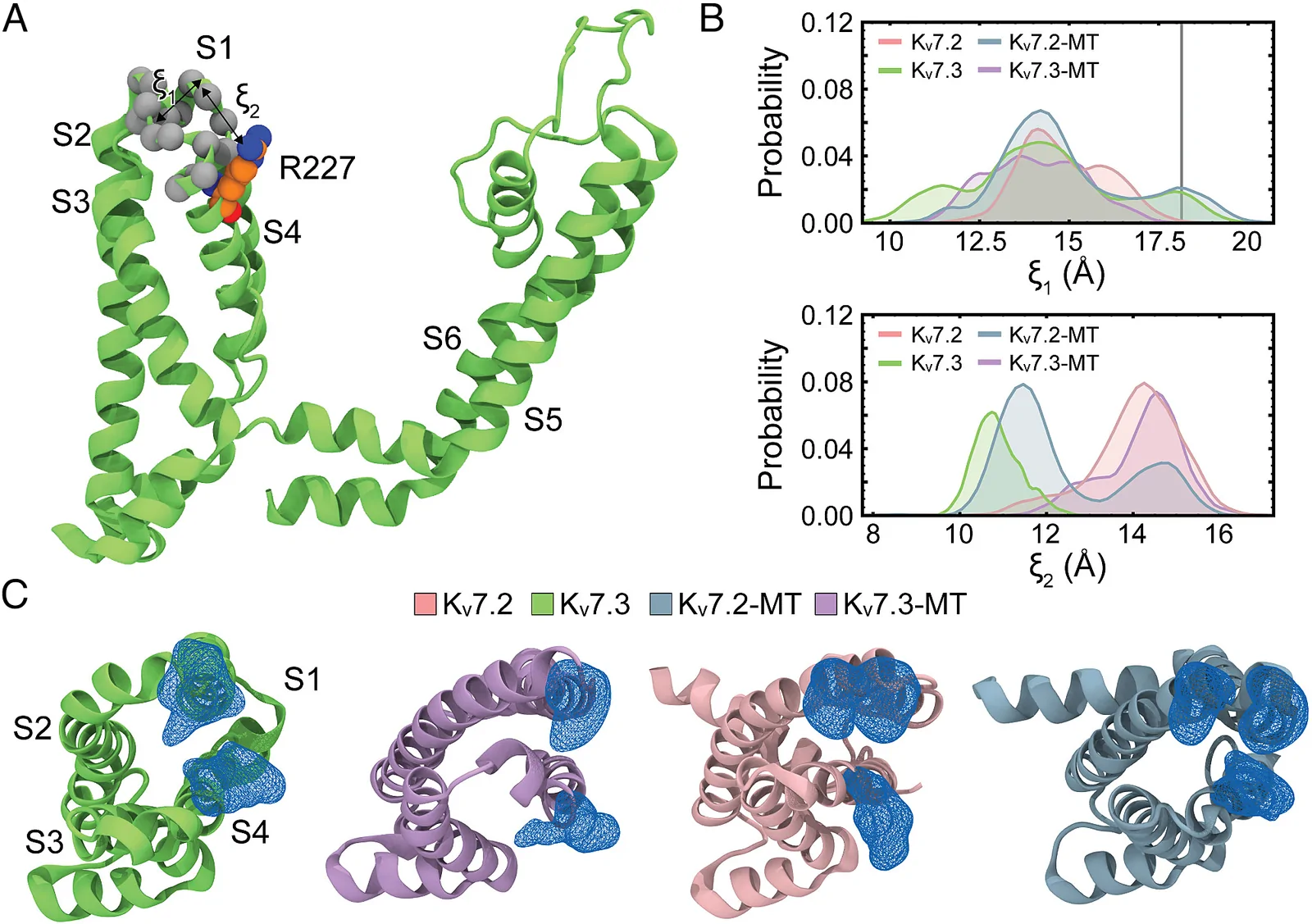
Mutations to the S3–S4 loop modulate accessibility and affinity of CA to site 3. (*A*) Kv7.3 monomer showing order parameters $\xi_{1}$ (distance between the S1–S2 and S3–S4 extracellular loops) and $\xi_{2}$ (distance between the S1–S2 loop and the cationic residue R227 on S4). Helices S1–S6 are labeled. (*B*) Probability distributions of $\xi_{1}$ (*Top*) and $\xi_{2}$ (*Bottom*) from MD simulations of the WT Kv channels and variants. $\xi_{1}=18\;Å$ is marked with a vertical gray line. (*C*) 3D density (ρ = 0.05) of the cationic residues lining site 3 in Kv7.3 (lime), Kv7.3-L222F + S225-A-L226 (violet), Kv7.2 (pink), and Kv7.2-A196del + F192L (light blue). Kv7.3 and the Kv7.2-A196del + F192L variant structures are taken from a state corresponding to the vertical line in panel *B*. In Kv7.3 and the Kv7.2-A196del + F192L variant, the cationic residue density remains concentrated in the interior of the VSD at site 3, whereas in Kv7.2 and Kv7.3-L222F + S225-A-L226, the density is redistributed and shifted away from the pocket.

Electrophysiological recordings demonstrate that transplanting the Kv7.3 S3–S4 loop onto Kv7.2 confers CA sensitivity (Fig. 7); conversely, transplanting the Kv7.2 S3–S4 loop into Kv7.3 reduces its sensitivity (Fig. 6). This pattern is consistent with VSD-targeted Kv7 potentiators, where subtype selectivity reflects how the VSD adopts binding-competent conformations set by S1–S2/S3–S4 elements (31, 32, 37, 41–43). To elucidate the mechanisms underlying this subtype-selective action, we performed molecular simulations of two S3–S4 loop modified variants: Kv7.3-L222F + S225-A-L226 and Kv7.2-A196del + F192L. We analyzed conformational changes in the VSD by defining two distance metrics. $\xi_{1}$ measures the separation between the S1–S2 and S3–S4 loops as a proxy for the opening of a site 3 binding pocket in the VSD (Fig. 8*A*), and $\xi_{2}$ measures the separation between the S1–S2 loop and a cationic residue (R227 in Kv7.3; R198 in Kv7.2) and describes how closely the cationic residue is positioned relative to the pocket entrance (Fig. 8*A*).

In simulations of both wild-type channels and their variants, the $\xi_{1}$ distributions had a maximal peak near 13 to 15 Å, indicating that the binding pocket maintains a similar baseline degree of opening across all constructs (Fig. 8*B*). Notably, the channels that are activated by CA, Kv7.3, and Kv7.2-A196del + F192L, also sampled a state with a wider site 3 pocket ($\xi_{1}$ = 18 Å). The $\xi_{2}$ distributions, on the other hand, were largely bimodal, with Kv7.3 and Kv7.2-A196del + F192L adopting shorter $\xi_{2}$ values ($\xi_{2}$ ~ 10 to 12 Å), placing a cationic residue on S4 proximal to the pocket entrance, whereas Kv7.2 and Kv7.3-L222F + S225-A-L226 had larger $\xi_{2}$ values ($\xi_{2}$ ~ 14 to 15 Å), corresponding to a displacement of the cationic residue away from site 3. We computed spatial densities for the extracellular cationic side chains lining site 3 (Fig. 8*C*). In constructs containing S3–S4 loops from WT Kv7.3 (Kv7.3 and Kv7.2-A196del + F192L) the cationic density is concentrated toward the interior of the VSD, consistent with a shorter $\xi_{2}$ value. In contrast, constructs containing S3–S4 loop sequences from WT Kv7.2 (Kv7.2 and Kv7.3-L222F + S225-A-L226) exhibit a redistributed cationic density that is shifted away from the pocket, mirroring their larger $\xi_{2}$ values. These results indicate that S3–S4 loop identity modulates ligand accessibility through steric hindrance and binding affinity via electrostatic interactions at site 3, providing a molecular basis for the subtype-selective action of CA.

### CA Synergizes with RTG to Rescue Seizures Through VSD–Pore Cooperativity.

**Fig. 9.**
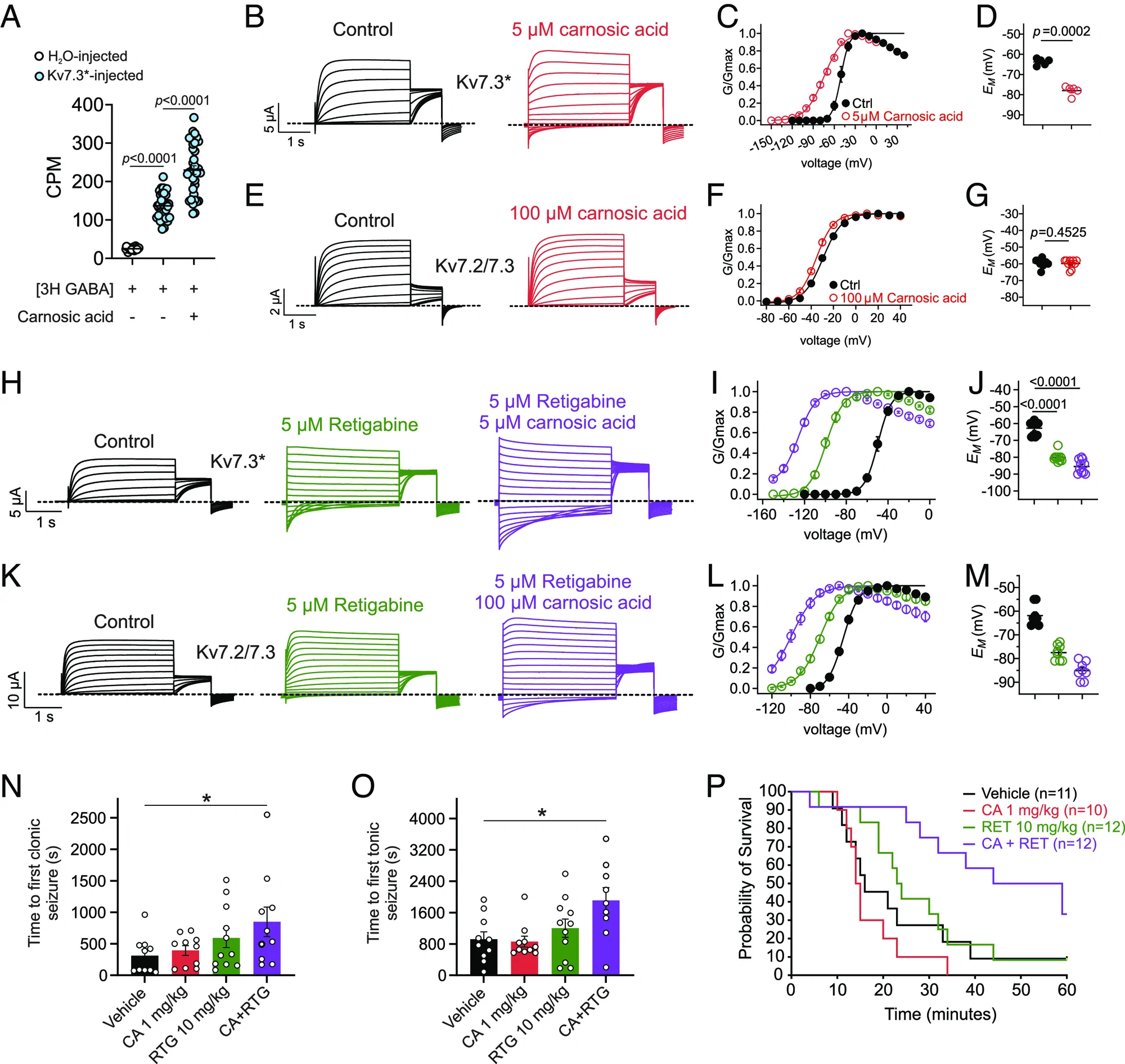
CA and RTG act at distinct binding sites to synergistically enhance Kv7.2/3 channel activity and reduce seizures. (*A*) Tritiated GABA binding assay for water-injected, Kv7.3*, and Kv7.3* and 100 µM CA in oocytes; *n* = 25 to 35. CPM, counts per minute. Assay conducted with two separate batches of oocytes. One-way ANOVA with Dunnett’s correction. (*B*) Mean traces for Kv7.3* in the absence (Control; black) or presence of 5 µM CA (*red*). Scale bars *Lower Left*; *n* = 5. (*C*) G/Gmax from *B*; *n* = 5. (*D*) Resting membrane potential from *B*; *n* = 5. Paired *t* test. (*E*) Mean traces for Kv7.2/3 in the absence (Control; *black*) or presence of 100 µM CA (*red*). Scale bars *Lower Left*; *n* = 10. (*F*) G/Gmax from *E*; *n* = 10. (*G*) Resting membrane potential as in *E*; *n* = 10. Paired *t* test. (*H*) Mean traces for Kv7.3* in the absence (Control; *black*) or presence of 5 µM retigabine (*green*) or 5 µM CA and 5 µM retigabine (*purple*); *n* = 10. Scale bars *Lower Left*. (*I*) G/Gmax from *H*; *n* = 10. (*J*) Resting membrane potential from *H*; *n* = 10. Paired *t* test. (*K*) Mean traces for Kv7.2/3 in the absence (Control; *black*) or presence of 5 µM retigabine (*green*) or 100 µM CA and 5 µM retigabine (*purple*); *n* = 8. Scale bars *Lower Left*. (*L*) G/Gmax from *K*; *n* = 8. (*M*) Resting membrane potential from *K*; *n* = 8. Paired *t* test. (*N*) Effects of vehicle, compared to 1 mg/kg CA, 10 mg/kg retigabine, or 1 mg/kg CA + 10 mg/kg RTG on time to first clonic seizure incidence; *n* = 10 to 11. *P = 0.0826. One-way ANOVA with Dunnett’s correction. (*O*) Effects of vehicle, compared to 1 mg/kg CA, 10 mg/kg retigabine, or 1 mg/kg CA + 10 mg/kg retigabine on time to first tonic seizure incidence; *n* = 10 to 11. *P = 0.0117. One-way ANOVA with Dunnett’s correction. (*P*) Seizure survival curve in a mouse PTZ chemoconvulsant model timed over 60 min for vehicle, 1 mg/kg CA, 10 mg/kg retigabine, or 1 mg/kg CA + 10 mg/kg RTG; *n* = 10 to 12.

Having established that CA stably associates with the S4 cap of the Kv7.3 voltage-sensing domain, we next asked whether occupancy of the CA site is pharmacologically independent of, or allosterically coupled to, ligands that bind the pore-adjacent site. To address this directly, we investigated the effect of CA on [^3^H]GABA binding to Kv7.3* and found CA did not prevent [^3^H]GABA binding, supporting our conclusion that the two ligands do not compete for the same binding site (4, 44) (Fig. 9*A*). Unexpectedly, CA instead enhanced [^3^H]GABA binding, suggesting positive allosteric coupling between the VSD-associated CA site and the canonical W265-dependent ligand pocket. This finding raised the possibility that CA might also potentiate the functional effects of other W265-dependent activators.

We decided to test whether CA could enhance the activity of the clinically relevant Kv7 opener RTG, which had been voluntarily withdrawn from the market in 2017 due to side effects including skin discoloration and retinal pigmentation changes. First, we screened 5 µM CA against Kv7.3*, which shifted V_0.5act_ −23.3 ± 3.1 mV and hyperpolarized the *E_M_* of unclamped oocytes (Fig. 9 *B*–*D* and *SI Appendix*, Table S20). We chose 5 µM as it enables us to assess the functional interaction of CA with RTG under submaximal conditions where additional potentiation could still be resolved. In contrast, we used 100 µM CA for Kv7.2/3 because it is largely insensitive to CA alone. As expected, 100 µM CA had a negligible effect at Kv7.2/3, shifting V_0.5act_ by −5.9 ± 0.6 mV and had no effect on the *E_M_* of unclamped oocytes (Fig. 9 *E*–*G* and *SI Appendix*, Table S21).

Retigabine (5 µM) robustly left-shifted the V_0.5act_ of Kv7.3* by −41.8 ± 1.9 mV, whereas coapplication of 5 µM CA further shifted the V_0.5act_ to −62.9 ± 1.1 mV. Both treatments produced similar changes in the *E_M_* of unclamped oocytes (Fig. 9 *H*–*J* and *SI Appendix*, Table S22). These results indicate that CA potentiates retigabine-mediated activation of Kv7.3*, consistent with positive allosteric communication between the VSD-associated CA site and the canonical W265-dependent ligand pocket. However, this effect appears additive rather than synergistic.

Retigabine and CA acid were additive at Kv7.3/5 heteromers. At 5 µM, CA shifted the V_0.5act_ of Kv7.3/5 −17 mV (*SI Appendix*, Fig. S13 *A*–*C* and Table S23), while retigabine shifted the V_0.5act_ −16 mV (*SI Appendix*, Fig. S13 *D*–*F* and Table S24). Coapplication of 5 µM CA and retigabine only hyperpolarized the V_0.5act_ by a further −9 mV (−25.5 ± 2.9 mV) (*SI Appendix*, Fig. S13 *D*–*F* and Table S24).

We next tested 5 µM RTG on Kv7.2/3 heteromers and observed a −24.0 ± 1.2 mV shift in V_0.5act_ accompanied by hyperpolarization of the *E_M_* of unclamped oocytes. Strikingly, coapplication of 100 µM CA produced a substantially larger effect, shifting V_0.5act_ to −53.4 ± 1.2 mV and further hyperpolarizing the *E_M_* of unclamped oocytes (Fig. 9 *K*–*M* and *SI Appendix*, Table S25). These results indicate that CA enhances retigabine-mediated activation of Kv7.2/3 to a degree greater than expected from its weak standalone effect, consistent with synergy.

Given our in vitro data unexpectedly predicted that CA and RTG synergize at Kv7.2/3, we next investigated whether this combination could have therapeutic activity in a mouse pentylenetetrazol (PTZ) chemoconvulsant assay. Neither CA (1 mg/kg) or RTG (10 mg/kg) had any effect on the time to first clonic or tonic seizure compared to vehicle (Fig. 9 *N* and *O*). However, when combined CA + RTG (1 mg/kg + 10 mg/kg) increased time to first clonic and tonic seizure almost twofold compared to vehicle (Fig. 9 *N* and *O*). Most strikingly, CA + RTG significantly reduced seizure-related mortality in mice, increasing probability of survival to 40% at 60 min compared to 10% for RTG alone (Fig. 9*P*). Together, these results demonstrate that CA and RTG synergistically activate Kv7.2/3 channels through allosteric communication between the VSD and pore domains, an interaction that reduces the time to first clonic and tonic seizures and decreases seizure-related mortality.

## Discussion

Our electrophysiological and MD analyses reveal that CA achieves Kv7.3 selectivity through differences in VSD conformational ensembles encoded by S4 cap architecture.

Quantitative analysis using the ξ_1_ and ξ_2_ order parameters demonstrates that although all Kv7 constructs tested maintain a similar baseline extracellular pocket separation (ξ_1_ ≈ 13 to 15 Å), only CA-responsive channels (Kv7.3 and Kv7.2-A196del + F192L) sample an expanded, binding-competent state (ξ_1_ ≈ 18 Å). Moreover, these constructs position a cationic S4 residue proximal to the pocket entrance (shorter ξ_2_), generating an electrostatically favorable microenvironment that stabilizes CA. In contrast, CA-insensitive constructs redistribute this charge away from the pocket, destabilizing ligand engagement. These conformational signatures align with electrophysiological gain- and loss-of-function mutations, establishing a direct link between S4 cap architecture, VSD conformational sampling, and isoform-selective activation.

Many Kv7 modulators act through the conserved S5 tryptophan-lined pocket, which has been extensively characterized (4, 5, 45–47). By contrast, our findings demonstrate that selective activation can arise from VSD-encoded conformational and electrostatic features independent of this canonical pore-binding site. Furthermore, our data identify L226 as a hydrophobic latch that regulates CA sensitivity by tuning the energetic landscape of the voltage sensor. In this framework, ligand efficacy is determined by the energetic accessibility of activation-competent states rather than binding alone. Substitutions that weaken the latch enhance CA-mediated activation, whereas substitutions that stabilize or disrupt the latch reduce or occlude CA efficacy.

Although there are other Kv7 modulators that target the VSD, their isoform selectivity has largely been attributed to local residue difference or inferred binding interactions, without a unified structural or mechanistic framework (31, 37, 41, 42, 47–50). Here, our data link isoform selectivity directly to VSD conformational sampling and electrostatic microenvironment, providing a quantitative mechanistic basis for selective channel activation.

Our previous work also demonstrates that ligand chemistry further shapes this selectivity. Notably, structurally related CA analogs lack activity at Kv7.3 (6), indicating a stringent requirement for chemical compatibility with the Kv7.3 channel. Together, these findings support a model in which isoform selectivity emerges from an interplay between ligand chemistry and the local VSD microenvironment.

For example, aloperine selectively activates Kv7.5 despite targeting a conserved VSD residue (44), indicating that the surrounding microenvironment is isoform-specific for functional engagement. Thus, conservation of a binding site alone is insufficient to predict pharmacological responsiveness without considering the surrounding conformational and electrostatic context.

A key finding of this study is that CA robustly potentiates retigabine-mediated activation of Kv7.2/3 despite lacking standalone activity at these channels. Together with enhanced GABA binding, this reveals positive allosteric coupling between the VSD-associated CA site and the canonical pore-adjacent ligand pocket. These findings demonstrate that VSD-directed ligands can allosterically prime channels to enhance pore-targeted activation. Given that retigabine was withdrawn from clinical use due to dose-limiting adverse effects, this mechanism suggests that VSD-targeted modulators could improve the safety profile of Kv7 activators by enabling dose reduction.

Together, these findings provide a framework for how isoform specificity can be encoded within highly conserved ion channel families. Isoform selectivity arises not from canonical binding determinants alone, but from isoform-specific differences that shape the conformational and electrostatic landscape of the VSD. More broadly, these results establish the voltage sensor as a tunable pharmacological scaffold whose conformational dynamics can be exploited to design next-generation isoform-selective modulators.

## Materials and Methods

A detailed description of materials and methods is provided in *SI Appendix*.

### cRNA Preparation, Injection, and Two Electrode Voltage-Clamp (TEVC).

cRNA preparation, *Xenopus laevis* oocyte injection and TEVC were performed as described before (6). Complete description available in *SI Appendix*.

### Radioligand Binding Competition Assays.

Radioligand binding assays were performed as described before (4). Complete description available in *SI Appendix*.

### In Silico Docking.

In silico docking was performed using DockThor (51). Complete description available in *SI Appendix*.

### All-Atom Models of Kv7.2 and Kv7.3 Channels.

The 3.1 Å cryo-EM structure of Kv7.2 (PDB: 7CR0) (31) was used as an initial model for simulations of Kv7.2 and Kv7.3 channels. A complete description of the models is provided in *SI Appendix*.

### MD Simulations of Kv7.2 and Kv7.3 Channels.

Simulations in the constant NPT ensemble were performed using a Nosé-Hoover thermostat at 310 K and a Nosé-Hoover Langevin barostat at 1 atm. Simulation setup and analysis of ligand density, residue–residue distances, dynamic cross correlation, and structural alignment are described in *SI Appendix*.

### Pentylenetetrazol-Induced Seizure Mouse Model.

The potential for in vivo synergy of retigabine with CA was examined in mice by combining subthreshold anticonvulsant doses of both compounds in a pentylenetetrazol-induced seizure model as described in *SI Appendix*.

### Statistics and Reproducibility.

All values are expressed as mean ± SEM. Statistics were conducted by using either a paired *t* test or Ordinary One-way ANOVA with Dunnett’s multiple comparison; all *P* values were two-sided. Electrophysiological data were confirmed in at least two batches of oocytes. Biological replicates are defined as numbers of oocytes; sample sizes are given in the figure legends.

## Supplementary Material

Appendix 01 (PDF)

Movie S1. Kv7.3 channel in the presence of six CA molecules. Kv7.3 is embedded in phospholipid bilayer and shown from a top and side view. Kv7.3 is colored in green. Carnosic acid stably binds to a cavity in the VSD.

Movie S2. Binding of CA to site 3 on Kv7.3. Close-up view of CA binding to the extracellular cavity of the VSD with interacting residues colored in orange. CA is shown in ball-and-stick representation.

Movie S3. The Kv7.2 channel in the presence of six CA molecules. Kv7.2 is embedded in a phospholipid bilayer and shown from a top and side view. Kv7.3 is colored in pink. Two binding/unbinding events of CA to/from Kv7.2 are shown.

Movie S4. Binding/unbinding of CA Kv7.2. Close-up view of CA binding/unbinding to the extracellular cavity of the VSD with interacting residues colored in orange. CA is shown in ball-and-stick representation. Binding site is equivalent to site 3 on Kv7.3.

Movie S5. The Kv7.3 channel with 12 molecules of carnosic acid bound to the three binding sites. Kv7.3 is embedded in phospholipid bilayer and shown from top and bottom view. Kv7.3 is colored in green. Three molecules of CA are placed at each of the three binding sites for each monomer of the channel.

## Data Availability

Source data (Electrophysiology data) for Figs. 1–9 are available at Dryad data repository (https://doi.org/10.5061/dryad.xsj3tx9xf) (52).
