# Supplementary material for: Voltage-sensor conformational microenvironments encode isoform-selective ion channel activation
Source: Proc Natl Acad Sci U S A. 2026 Sep 23;123(39):e2616367123. doi: 10.1073/pnas.2616367123 (PMC13624614; doi:10.1073/pnas.2616367123)
Supplement: Supplementary file 1 [file pnas.2616367123.sapp.pdf]

Supporting Information for

Voltage-sensor conformational microenvironments encode isoform-selective ion channel activation

Rían W. Manville[1*=], Lorenzo Foglia[2=], Ryan Yoshimura[1], Daniel E. Santos[2], Alvin Yu[2*], Geoffrey W. Abbott[1*]

[1]Bioelectricity Laboratory, Dept. of Physiology and Biophysics, School of Medicine, University of California, Irvine, CA, USA.

[2]Yu Lab, Dept. of Physiology and Biophysics, School of Medicine, University of California, Irvine, CA, USA.

*Corresponding Authors

Geoffrey W. Abbott (abbottg@hs.uci.edu)

Alvin Yu (alviny6@uci.edu)

Rían W. Manville (rmanvill@uci.edu)

This PDF file includes

Detailed materials and methods

Captions for Movies S1 to S5

Supplementary Information (SI) Tables 1 to 25

Supplementary Information (SI) Appendix Fig. S1 to S13

SI References

Other supplementary materials for this manuscript include the following:

Movies S1 to S5

Materials and methods

cRNA preparation and *Xenopus laevis* oocyte injection

We generated cRNA transcripts encoding human Kv7.2 and Kv7.3 (wild-type and mutant) by *in vitro* transcription using the mMessage mMachine kit (Thermo Fisher Scientific, Waltham, MA, USA) according to the manufacturer's instructions, after vector linearization, from cDNA sub-cloned into expression vectors (pTLNx and pXOOM) incorporating *Xenopus laevis* β-globin 5' and 3' UTRs flanking the coding region to enhance translation and cRNA stability. Mutant Kv7 cDNAs were generated by Genscript (Piscataway, NJ, USA) and cRNAs made as above. We injected defolliculated stage V and VI *Xenopus laevis* oocytes (Xenoocyte, Dexter, MI, USA) with Kv7 cRNAs (2-10 ng) and incubated the oocytes at 16 ^{o}C in Barth's solution oocyte storage solution containing penicillin and streptomycin, with daily washing, for 2-4 days prior to two-electrode voltage clamp (TEVC) recording.

Two-electrode voltage clamp (TEVC)

We performed TEVC at room temperature using an OC-725C amplifier (Warner Instruments, Hamden, CT, USA) and pClamp10 software (Molecular Devices, Sunnyvale, CA, USA) 2-4 days after cRNA injection. We placed oocytes in a small-volume oocyte bath (Warner) and viewed them with a dissection microscope for cellular electrophysiology. We sourced chemicals from Sigma-Aldrich (St. Louis, MO, USA) and Combi-Blocks (San Diego, CA, USA). We studied the effects of compounds solubilized directly in bath solution (in mM): 96 NaCl, 4 KCl, 1 $MgCl_2$, 1 $CaCl_2$, 10 HEPES (pH 7.6). We introduced compounds into the oocyte recording bath by gravity perfusion at a constant flow of 1 ml per minute for 3 minutes prior to recording. Pipettes were of 1-2 MΩ resistance when filled with 3 M KCl. We recorded currents in response to voltage pulses between -150 mV, -120 mV or -80 mV and +40 mV at 10 mV intervals, followed by a tail pulse to -30 mV, from a holding potential of -80 mV, to yield current-voltage relationships. We analyzed data using Clampfit (Molecular Devices) and Graphpad Prism software (GraphPad, San Diego, CA, USA), stating values as mean ± SEM. We plotted raw or normalized tail currents versus prepulse voltage and fitted with a single Boltzmann function:

Eq. 1

$$g = A1 + \frac{(A_2 - A_1)}{\left\{1 + exp\,[V - V_{\frac{1}{2}}]/Vs\right\}}$$

where g is the normalized tail conductance, A_1 is the initial value at -∞, A_2 is the final value at +∞, $V_{1/2}$ is the half-maximal voltage of activation and V_s the slope factor. We fitted activation and deactivation kinetics with single exponential functions.

In silico docking

We performed unguided docking to predict interaction sites for retigabine, gabapentin, and GABA using the neuronal Kv7 (Kv7.3) model structure and the receptor-ligand screening platform DockThor[51]. For *in silico* determination of the binding site predictions of CA on Kv7.3 we used docking algorithms with SwissDock with CHARMM forcefields[1, 33] and machine-learning based techniques based on statistics from the Protein Data bank, i.e. AlphaFold[34]. The chemical structure of CA was defined using its SMILES representation and used as the ligand. Docking was performed using the SwissDock and AlphaFold web servers. The Kv7.3 channel structure and the SMILES sequence of the ligand were provided as inputs to SwissDock, while Kv7.3 amino acid sequence and the SMILES sequence of the ligand were provided to AlphaFold. The docking poses were analyzed using VMD.

All-atom models of Kv7.2 and Kv7.3 channels

The 3.1 Å cryo-EM structure of native Kv7.2 (PDB: 7CR0)[31] was used as an initial model for simulations of this channel. Missing loop regions were modeled using AlphaFold[34]. The mutant Kv7.2 was derived from the structure by introducing a leucine substitution at residue 192 and an alanine deletion at residue 196 in each subunit using PyMol[2]. As no experimental structure was available for Kv7.3 the predicted structure of the Kv7.3 monomer available in the AlphaFold database[34] (UNIPROT: AF-O43525-F1) was used as an initial model. Transmembrane segments of Kv7.3 including the S1–S6 helices were retained, and disordered, intracellular regions (residues 1-109 and 370-872) were removed. The cryo-EM structure for

the Kv7.2 tetramer (PDB ID: 7CR0)[31] was used as a template to obtain the quaternary protein-protein contacts that stabilize Kv7.3. Alignment was performed by minimizing the root-mean-square deviation (RMSD) between the protein backbone of the S5 and S6 helices (resid: 258-284 and 327-369) in the Kv7.3 and the protein backbone of the S5 and S6 helices (resid: 167-193 and 226-268) in Kv7.2 to preserve the protein–protein contacts found in the tetramer and the overall conformation of the pore and selectivity filter. Structural alignment was carried out using the Visual Molecular Dynamics (VMD) software package[3]. The Kv7.3 variant was derived from the WT structure by introducing a phenylalanine substitution at residue 222 and an alanine insertion between residues 225-226 of each subunit using PyMol software[2].

For all channels, an asymmetric lipid bilayer with a lipid species composition found in neurons[4] consisting of 47% cholesterol (CHOL), 14% 1-palmitoyl-2-oleoylphosphatidylethanolamine (POPE), 27% 1-palmitoyl-2-oleoyl-sn-glycero-3-phosphocholine (POPC), and 12% sphingomyelin (SM) in the upper leaflet and 47% CHOL, 10% 1-palmitoyl-2-oleoyl-sn-glycero-3-phospho-L-serine (POPS), 22% POPE, 14% POPC, 5% 1-palmitoyl-2-oleoyl-sn-glycero-3-phosphoinositol (POPI) and 2% SM molecules in the lower leaflet was constructed using CHARMM-GUI[5,6]. The resulting lipid bilayer contained a total of 400 lipid molecules. All channels were embedded into the lipid bilayer using the protein replacement method[7]. For WT Kv7.3 and WT Kv7.2 models, six deprotonated CA ligands were added into the bulk solution at least 10 Å away from any non-ligand molecules and solvated with 53952 and 45177 water molecules, respectively. The models for WT Kv7.3 without CA, L222F + S225-A-L226 Kv7.3, WT Kv7.2 without CA, and A196del + F192L Kv7.2, were solvated with 27585, 40385, 43846, and 43884, respectively. 150 mM KCl were added to bulk solvent, and ion concentrations were adjusted to maintain electrostatically neutral systems.

MD simulations of Kv7.2 and Kv7.3 channels

The solvated WT Kv7.3 + CA, WT Kv7.3, L222F + S225-A-L226 Kv7.3, WT Kv7.2 + CA, WT Kv7.2, and A196del + F192L Kv7.2 systems contained a total number of atoms ranging from 125,439 to 221,920. Periodic boundary conditions were imposed on orthorhombic unit cells of dimensions ranging from (112, 116, 146) Å to (135, 135, 245) Å in the (x,y,z) directions, respectively. The all-atom potential energy function CHARMM36m[8,9] for proteins, membrane, and ions and the TIP3P[10] potential energy function for water were used. CA molecules were parameterized using the CHARMM General Force Field (CGenFF)[11]. The all-atom systems were energy minimized and then equilibrated under constant pressure and temperature (NPT) conditions. Simulations in the constant NPT ensemble were performed using a Nosé-Hoover thermostat at 310 K and a Nosé-Hoover Langevin barostat at 1 atm. Bond-lengths for hydrogen atoms were constrained using the SHAKE algorithm[12]. An r-RESPA integrator was used with a timestep of 2 fs; long-range electrostatics were computed every 2 fs[13]. Long-range electrostatics were calculated using the particle mesh Ewald algorithm, and short-ranged, nonbonded interactions were truncated at 12 Å[14]. All simulations used the all-atom MD simulation packages NAMD 2.14 and NAMD 3.0[15]. For WT Kv7.3 + CA, production-level runs were performed on five replicates. In total, the three systems were simulated for 218 ns, 198 ns, and 168 ns for an aggregate simulation time of 584 ns. For WT Kv7.3, L222F + S225-A-L226 Kv7.3, WT Kv7.2 + CA, WT Kv7.2, and A196del + F192L Kv7.2 systems, production-level runs were performed on one initial condition per system containing the respective channel, a lipid bilayer, and bulk solvent. In total, the four systems were simulated for 212 ns, 269 ns, 218 ns, 206 ns, and 229 ns, respectively. For all subsequent analysis all-atom trajectories were sampled at 0.02-ns intervals. These MD simulations were performed in the presence of an externally applied electric field with constant electric field strength[25] of −0.5 mV/Å or −0.012 kcal/(mol Å e). To evaluate the stability of the ligand at the binding site, we also simulated WT Kv7.3 with one CA molecule bound to the binding site of each monomer (twelve total). Additional MD simulations of

Kv7.3 in the open state with six molecules of CA in bulk solvent were performed to evaluate the state-dependency of binding. Kv7.3 in the open conformation was obtained by aligning the previously developed model of Kv7.3 to an available structure of the channel in the open state (PDB ID: 22RQ) using the selectivity filter as reference for alignment (resid > 315 and resid < 323). The channel was embedded in phospholipid bilayer and solvated, and neutralized in a orthorhombic cell of (150, 150, 150) Å as previously described.

Ligand density

To compute the ligand density maps of CA on Kv7.3, we averaged five replicas of a system with Kv7.3 embedded in a phospholipid membrane and six molecules of CA in bulk solvent. For each frame of the trajectories, the atomic coordinates were aligned to the initial structure by minimizing the root-mean-square deviation (RMSD) of the protein backbone atoms with respect to their starting positions[16,26]. Following alignment, the Cartesian coordinates of all non-hydrogen atoms of CA were recorded, and the three-dimensional spatial density of atomic positions, ρ(r), was computed. The density was evaluated on a discretized three-dimensional grid with a spacing of 0.5 Å × 0.5 Å × 0.5 Å, treating each atom using a hard sphere van der Waals approximation. The resulting 3D ligand density map was visualized by contouring $\rho(r)$ at isovalues of 0.22, 0.11, and 0.02, with ρ = 0.44 representing the maximum density observed. These contour levels, corresponding to progressively lower occupancy thresholds, were rendered on the Kv7.3 ion channel to identify and compare the most frequently sampled positions and preferred binding regions of CA. The same approach was used to compute the density of cationic residues on WT Kv7.3, L222F + S225-A-L226 Kv7.3, WT Kv7.2, and A196del + F192L Kv7.2. For these density maps, ρ(r) was contoured with an isovalue of 0.05.

Structural alignment

To compare ligand stability at each binding site, we analyzed a trajectory containing the Kv7.3 embedded in a phospholipid membrane with twelve bound ligands: three per channel subunit, occupying all three

distinct binding sites. The entire trajectory was aligned to its first frame, with a local alignment performed on the monomer bearing the bound ligand of interest. Using this procedure, we quantified the motion of each ligand relative to its initial position using the root-mean-square deviation (RMSD). To evaluate the structural changes induced by CA on Kv7.3, we examined two separate simulations: one with CA present in the bulk solvent and one without CA. For both simulations, the first seventy modeled residues of Kv7.3 were aligned at the beginning of the simulation and after 200 ns. To evaluate the structural similarity between the VSD in open, closed and CA-bound states, we RMSD aligned residues 154–169 of the S2 helix.

Distances calculation

To quantify the conformational changes induced by the ligand upon binding on WT-Kv7.3, we measured the Euclidean distance between the centers of mass of the S4-S5 linker (residues 244-255), and the angles formed by the third principal axis of inertia of S3 (residues 197-215) and S4 (residues 225-238) in the bound and apo states. Two one-dimensional order parameters (ξ_1 and ξ_2) are used to quantify the conformational rearrangements of the VSD. Each order parameter is defined as a center-of-mass distance between specified elements of the VSD. ξ_1 measures the separation between the S1-S2 loop and the S3-S4 loop. For Kv7.3, ξ_1 is defined as the distance between the center-of-mass of residues: 139-149, (S1-S2) and the center-of-mass of residues: 217-223 (S3-S4). For Kv7.2, ξ_1 is the distance between the center-of-mass of residues: 106-116 (S1-S2) and the center-of-mass of residues 187-193 (S3-S4). ξ_2 measures the separation between the S1-S2 loop and a cationic residue at the mouth of the extracellular pocket in S4. ξ_2 is defined as the center-of-mass distance between the S1-S2 loop and (R227 in Kv7.3 and R198 in Kv7.2). For each trajectory (Kv7.2, Kv7.3, Kv7.2-A196del + F192L, and Kv7.3-L222F + S225-A-L226), ξ_1 and ξ_2 were evaluated at 0.08 ns intervals across all four subunits, and the resulting values were pooled to generate one distribution per subtype. Probability distributions were computed by binning

the pooled values into 50 equally spaced bins spanning the full observed range and normalizing by the total number of samples.

Dynamic cross correlation

We computed dynamic cross correlation matrices[16] (DCCM) for the systems in which CA binds to Kv7.3, the system with Kv7.3 initiated from the closed state and the system with Kv7.3 initiated from the open state. We computed the DCCM of the Cα movement for every residue in the VSD of Kv7.3 in the three systems for one monomer of the channel, considering the CA-bound monomer for the system in the bound state. Cross correlation values for any pair (i, j) of two residues were computed as:

$$C_{ij} = \frac{\langle \Delta r_i(t) \cdot \Delta r_j(t) \rangle_t}{\sqrt{\langle \|\Delta r_i(t)\|^2 \rangle_t \langle \|\Delta r_j(t)\|^2 \rangle_t}}$$

Where $r_i(t)$ is the position of the Cα atom of residue i, $\Delta r_i(t) = r_i(t) - \langle r_i(t) \rangle_t$ is the fluctuation of the position with respect to its time average.

Pentylenetetrazol-induced seizure mouse model

Adult, male C57BL/6 mice (Charles River, Wilmington, MA) were group housed under a 12-hour light: dark cycle and given access to food and water *ad libitum*. The mice tested were between 7 and 13 weeks of age. This study was performed under an approved Institutional Animal Care and Use Committee protocol at the University of California, Irvine.

1 mg/mL of CA (Combi-Blocks, San Diego, CA), 10 mg/mL of free base retigabine dihydrochloride (Alomone Labs, Jerusalem, Israel), or a combination of the two were solubilized in DMSO. PTZ (Sigma-Aldrich, St. Louis, MO) was made up at 9 mg/mL in PBS.

Each animal initially received a 1 mL/kg intraperitoneal injection of vehicle, CA alone (1 mg/kg), retigabine alone (10 mg/kg), or CA and retigabine (1 mg/kg and 10 mg/kg, respectively). 30 minutes after the initial injection, each animal received a 10 mL/kg intraperitoneal injection of PTZ (90 mg/kg) and was placed in an empty home cage for observation for at least 60 minutes. The time and number of each occurrence of clonic and tonic seizures was recorded. The time of death was also recorded. Unless otherwise noted, all animals that survived for at least 60 minutes were also alive and appeared healthy at 24 hours.

Supplementary Movie 1. Kv7.3 channel in the presence of six CA molecules. Kv7.3 is embedded in phospholipid bilayer and shown from a top and side view. Kv7.3 is colored in green. Carnosic acid stably binds to a cavity in the VSD.

Supplementary Movie 2. Binding of CA to site 3 on Kv7.3. Close-up view of CA binding to the extracellular cavity of the VSD with interacting residues colored in orange. CA is shown in ball-and-stick representation.

Supplementary Movie 3. The Kv7.2 channel in the presence of six CA molecules. Kv7.2 is embedded in a phospholipid bilayer and shown from a top and side view. Kv7.3 is colored in pink. Two binding/unbinding events of CA to/from Kv7.2 are shown.

Supplementary Movie 4. Binding/unbinding of CA Kv7.2. Close-up view of CA binding/unbinding to the extracellular cavity of the VSD with interacting residues colored in orange. CA is shown in ball-and-stick representation. Binding site is equivalent to site 3 on Kv7.3.

Supplementary Movie 5. The Kv7.3 channel with 12 molecules of carnosic acid bound to the three binding sites. Kv7.3 is embedded in phospholipid bilayer and shown from top and bottom view. Kv7.3 is colored in green. Three molecules of CA are placed at each of the three binding sites for each monomer of the channel.

Supplementary Information (SI) Tables 1-25

Kv7.3*	$V_{0.5}$ Normalized tail current (mV)
Control	-50.28 ± 3.09 (*n* = 5)
0.1 µM Carnosic acid	-54.91 ± 1.90 (*n* = 5; *p*=0.9737)
1 µM Carnosic acid	-60.08 ± 1.35 (*n* = 5; *p*=0.6083)
3 µM Carnosic acid	-68.66 ± 3.48 (*n* = 5; *p*=0.0884)
10 µM Carnosic acid	-83.16 ± 8.21 (*n* = 5; *p*=0.0008)
30 µM Carnosic acid	-103.96 ± 8.86 (*n* = 5; *p*<0.0001)
100 µM Carnosic acid	-124.18 ± 4.63 (*n* = 5; *p*<0.0001)

Supplementary Table 1: Kv7.3* in the absence or presence of 0.1-100 µM carnosic acid. Values indicate mean ± SEM. Statistics conducted using an ordinary One-way ANOVA with Dunnett's multiple comparison test.

	$V_{0.5}$ Normalized tail current (mV)
Kv7.3*-V221A	-77.96 ± 2.69 (*n* = 10)
Kv7.3*-L222A	-78.41 ± 2.99 (*n* = 9)
Kv7.3*-T224A	-50.43 ± 1.83 (*n* = 8)
Kv7.3*-S225A	-30.79 ± 1.39 (*n* = 9)
Kv7.3*-L226A	-47.65 ± 0.72 (*n* = 17)
Kv7.3*-R227A	-56.80 ± 0.86 (*n* = 9)
Kv7.3*-S228A	-61.11 ± 2.10 (*n* = 7)
Kv7.3*-L229A	-31.29 ± 2.18 (*n* = 10)
Kv7.3*-R230A	*NA*
Kv7.3*-F231A	-55.97 ± 3.31 (*n* = 8)
Kv7.3*-L232A	-35.91 ± 1.51 (*n* =9)
Kv7.3*-Q233A	-48.48 ± 1.70 (*n* = 9)
Kv7.3*-I234A	*NF*
Kv7.3*-L235A	-40.89 ± 1.64 (*n* = 13)
Kv7.3*-R236A	-26.30 ± 1.98 (*n* = 18)
Kv7.3*-M237A	*NF*
Kv7.3*-L238A	-38.71 ± 0.83 (*n* = 10)
Kv7.3*-R239A	*NF*
Kv7.3*-M240A	-14.62 ± 0.49 (*n* = 10)
Kv7.3*-D241A	*NF*
Kv7.3*-R242A	-10.19 ± 1.88 (*n* = 11)
Kv7.3*-R243A	-32.20 ± 1.54 (*n* = 9)

Supplementary Table 2: Kv7.3*mutants baseline voltage-dependence of activation ($V_{0.5activation}$) in 4K. *NA* = not applicable; *NF* = non-functional. Values indicate mean ± SEM.

	Control $V_{0.5activation}$ (mV)	10 µM carnosic acid $V_{0.5activation}$ (mV)
Kv7.3*-V221A	-77.96 ± 2.69 (*n* = 10)	-96.61 ± 1.11 (*n* = 10; *p*<0.0001)
Kv7.3*-L222A	-78.41 ± 2.99 (*n* = 9)	-87.19 ± 2.76 (*n* = 9; *p*=0.0017)
Kv7.3*-T224A	-50.43 ± 1.83 (*n* = 8)	-83.68 ± 4.30 (*n* = 8; *p*<0.0001)
Kv7.3*-S225A	-30.79 ± 1.39 (*n* = 9)	-45.56 ± 1.57 (*n* = 9; *p*<0.0001)
Kv7.3*-L226A	-47.65 ± 1.11 (*n* = 17)	-136.96 ± 1.24 (*n* = 17; *p*<0.0001)
Kv7.3*-R227A	-56.80 ± 0.86 (*n* = 9)	-81.87 ± 3.42 (*n* = 9; *p*=0.0001)
Kv7.3*-S228A	-61.11 ± 2.10 (*n* = 7)	-68.07 ± 1.94 (*n* = 7; *p*=0.0120)
Kv7.3*-L229A	-31.29 ± 2.18 (*n* = 10)	-84.64 ± 1.86 (*n* = 10; *p*<0.0001)
Kv7.3*-F231A	-55.97 ± 3.31 (*n* = 8)	-71.63 ± 2.57 (*n* = 8; *p*=0.0002)
Kv7.3*-L232A	-35.91 ± 1.51 (*n* =9)	-58.28 ± 1.08 (*n* = 9; *p*<0.0001)
Kv7.3*-Q233A	-48.48 ± 1.70 (*n* = 9)	-79.96 ± 6.26 (*n* = 9; *p*=0.0026)
Kv7.3*-L235A	-40.89 ± 1.64 (*n* = 13)	-58.34 ± 2.08 (*n* = 13; *p*<0.0001)
Kv7.3*-R236A	-26.30 ± 1.98 (*n* = 18)	-34.36 ± 1.36 (*n* = 18; *p*<0.0001)
Kv7.3*-L238A	-38.71 ± 0.83 (*n* = 10)	-68.62 ± 2.43 (*n* = 10; *p*<0.0001)
Kv7.3*-M240A	-14.62 ± 0.49 (*n* = 10)	-31.34 ± 0.96 (*n* = 10; *p*<0.0001)
Kv7.3*-R242A	-10.19 ± 1.88 (*n* = 11)	-23.00 ± 1.25 (*n* = 11; *p*<0.0001)
Kv7.3*-R243A	-32.20 ± 1.54 (*n* = 9)	-47.11 ± 1.92 (*n* = 9; *p*<0.0001)

Supplementary Table 3: Kv7.3* mutants in the absence or presence of 10 µM carnosic acid. Values indicate mean ± SEM. Statistics conducted using a paired *t*-test.

Kv7.3*-R236A	$V_{0.5}$ Normalized tail current (mV)
Control	-24.15 ± 3.38 (*n* = 6)
0.1 µM Carnosic acid	-28.52 ± 1.57 (*n* = 6; *p*=0.1009)
1 µM Carnosic acid	-31.53 ± 0.60 (*n* = 6; *p*=0.0204)
3 µM Carnosic acid	-33.31 ± 0.27 (*n* = 6; *p*=0.0032)
10 µM Carnosic acid	-36.23 ± 0.56 (*n* = 6; *p*=0.0001)
30 µM Carnosic acid	-38.18 ± 1.43 (*n* = 6; *p*<0.0001)
100 µM Carnosic acid	-42.10 ± 1.86 (*n* = 6; *p*<0.0001)

Supplementary Table 4: Kv7.3*-R236A in the absence or presence of 0.1-100 µM carnosic acid. Values indicate mean ± SEM. Statistics conducted using an ordinary One-way ANOVA with Dunnett's multiple comparison test.

Kv7.3*-L226A	$V_{0.5}$ Normalized tail current (mV)
Control	-50.43 ± 2.99 (*n* = 7)
0.01 µM Carnosic acid	-57.34 ± 2.79 (*n* = 7; *p*=0.2433)
0.1 µM Carnosic acid	-66.12 ± 3.21 (*n* = 7; *p*=0.0034)
0.3 µM Carnosic acid	-80.41 ± 3.98 (*n* = 7; *p*<0.0001)
1 µM Carnosic acid	-107.35 ± 4.31 (*n* = 7; *p*<0.0001)
3 µM Carnosic acid	-125.36 ± 2.63 (*n* = 7; *p*<0.0001)
10 µM Carnosic acid	-142.20 ± 2.12 (*n* = 7; *p*<0.0001)
100 µM Carnosic acid	-165.76 ± 5.71 (*n* = 7; *p*<0.0001)

Supplementary Table 5: Kv7.3*-L226A in the absence or presence of 0.01-100 µM carnosic acid. Values indicate mean ± SEM. Statistics conducted using an ordinary One-way ANOVA with Dunnett's multiple comparison test.

**Kv7.3*-L226V**	**$V_{0.5}$ Normalized tail current (mV)**
Control	-54.68 ± 1.53<br>(*n* = 8)
10 μM Carnosic acid	-97.14 ± 3.48<br>(*n* = 8; *p*<0.0001)

Supplementary Table 6: Kv7.3*-L226V in the absence or presence of 10 μM carnosic acid. Values indicate mean ± SEM. Statistics conducted using a paired *t*-test.

**Kv7.3*-L226F**	**$V_{0.5}$ Normalized tail current (mV)**
Control	-62.80 ± 2.10<br>(*n* = 10)
10 μM Carnosic acid	-97.03 ± 1.82<br>(*n* = 10; *p*<0.0001)

Supplementary Table 7: Kv7.3*-L226F in the absence or presence of 10 μM carnosic acid. Values indicate mean ± SEM. Statistics conducted using a paired *t*-test.

**Kv7.3*-L226W**	**$V_{0.5}$ Normalized tail current (mV)**
Control	-69.44 ± 0.93<br>(*n* = 7)
10 μM Carnosic acid	-108.01 ± 0.61<br>(*n* = 7; *p*<0.0001)

Supplementary Table 8: Kv7.3*-L226W in the absence or presence of 10 μM carnosic acid. Values indicate mean ± SEM. Statistics conducted using a paired *t*-test.

**Kv7.3*-L226S**	**$V_{0.5}$ Normalized tail current (mV)**
Control	-105.26 ± 2.81<br>(*n* = 10)
10 μM Carnosic acid	*N.A.*

Supplementary Table 9: Kv7.3*-L226S in the absence or presence of 10 μM carnosic acid. Values indicate mean ± SEM. *N.A.* = not applicable.

Kv7.3*-L226Q	$V_{0.5}$ Normalized tail current (mV)
Control	-64.44 ± 2.79 (*n* = 10)
10 μM Carnosic acid	*N.A.*

Supplementary Table 10: Kv7.3*-L226Q in the absence or presence of 10 μM carnosic acid. Values indicate mean ± SEM. *N.A.* = not applicable.

Kv7.3*-S225-‘A’-L226	$V_{0.5}$ Normalized tail current (mV)
Control	-66.24 ± 2.71 (*n* = 8)
100 μM Carnosic acid	-74.29 ± 1.26 (*n* = 8; *p*=0.0211)

Supplementary Table 11: Kv7.3*-S225-‘A’-L226 in the absence or presence of 100 μM carnosic acid. Values indicate mean ± SEM. Statistics conducted using a paired *t*-test.

Kv7.3*-L222F S225-‘A’-L226	$V_{0.5}$ Normalized tail current (mV)
Control	-65.83 ± 1.04 (*n* = 7)
100 μM Carnosic acid	-66.62 ± 1.06 (*n* = 7; *p*=0.0310)

Supplementary Table 12: Kv7.3*-L222F-S225-‘A’-L226 in the absence or presence of 100 μM carnosic acid. Values indicate mean ± SEM. Statistics conducted using a paired *t*-test.

	Peak current (μA)
Kv7.2	1.32 ± 0.10 (*n* = 7)
Kv7.2-F192L	0.08 ± 0.01 (*n* = 6; *p*<0.0001)
Kv7.2-A196del	0.08 ± 0.01 (*n* = 7; *p*<0.0001)
Kv7.2-F192L, A196del	0.09 ± 0.01 (*n* = 7; *p*<0.0001)

Supplementary Table 13: Kv7.2 in the absence or presence of 100 μM carnosic acid. Values indicate mean ± SEM. Statistics conducted using a paired *t*-test.

	Peak current (μA)
Kv7.2/3	16.65 ± 1.24 (*n* = 8)
Kv7.3	0.24 ± 0.01 (*n* = 10)
Kv7.2-F192L/7.3	2.33 ± 0.38 (*n* = 8; *p*<0.0001)
Kv7.2-A196del/7.3	2.09 ± 0.31 (*n* = 8; *p*<0.0001)
Kv7.2-F192L, A196del/7.3	1.86 ± 0.20 (*n* = 9; *p*<0.0001)

Supplementary Table 14: Kv7.2/3 wild-type, Kv7.3 wild-type, Kv7.2-F192L/7.3, Kv7.2-A196del/7.3, and Kv7.2-F192L, A196del/7.3 baseline peak current in 4K. Values indicate mean ± SEM. Statistics conducted using a paired *t*-test.

	$V_{0.5}$ Normalized tail current (mV)
Kv7.2/3	-29.70 ± 0.47 (*n* = 8)
Kv7.2-F192L/7.3	-42.00 ± 1.99 (*n* = 8; *p*<0.0001)
Kv7.2-A196del/7.3	-26.34 ± 1.18 (*n* = 8; *p*=0.0067)
Kv7.2-F192L, A196del/7.3	-21.86 ± 1.86 (*n* = 8; *p*=0.0067)

Supplementary Table 15: Kv7.2/3 wild-type, Kv7.2-F192L/7.3, Kv7.2-A196del/7.3, and Kv7.2-F192L, A196del/7.3 baseline voltage-dependence of activation ($V_{0.5activation}$) in 4K. Values indicate mean ± SEM. Statistics conducted using a paired *t*-test.

Kv7.2/3	$V_{0.5}$ Normalized tail current (mV)
Control	-29.70 ± 0.47 (*n* = 8)
100 μM Carnosic acid	-38.84 ± 1.41 (*n* = 8; *p*=0.0002)

Supplementary Table 16: Kv7.2/3 in the absence or presence of 100 μM carnosic acid. Values indicate mean ± SEM. Statistics conducted using a paired *t*-test.

**Kv7.2-F192L/7.3**	**$V_{0.5}$ Normalized tail current (mV)**
Control	-42.00 ± 1.99 (*n* = 8)
100 µM Carnosic acid	-62.36 ± 3.08 (*n* = 8; *p*=0.0001)

Supplementary Table 17: Kv7.2-F192L/7.3 in the absence of or presence of 100 µM carnosic acid. Values indicate mean ± SEM. Statistics conducted using a paired *t*-test.

**Kv7.2-A196del/7.3**	**$V_{0.5}$ Normalized tail current (mV)**
Control	-26.34 ± 1.18 (*n* = 8)
100 µM Carnosic acid	-51.04 ± 0.81 (*n* = 8; *p*<0.0001)

Supplementary Table 18: Kv7.2-A196del/7.3 in the absence or presence of 100 µM carnosic acid. Values indicate mean ± SEM. Statistics conducted using a paired *t*-test.

**Kv7.2-F192L, A196del/7.3**	**$V_{0.5}$ Normalized tail current (mV)**
Control	-21.86 ± 1.86 (*n* = 8)
100 µM Carnosic acid	-58.19 ± 3.50 (*n* = 8; *p*<0.0001)

Supplementary Table 19: Kv7.2-F192L, A196del in the absence of or presence of 100 µM carnosic acid. Values indicate mean ± SEM. Statistics conducted using a paired *t*-test.

**Kv7.3***	**$V_{0.5}$ Normalized tail current (mV)**
Control	-50.58 ± 2.09 (*n* = 5)
5 µM Carnosic acid	-73.91 ± 1.78 (*n* = 5; *p*<0.0001)

Supplementary Table 20: Kv7.3* in the absence of or presence of 5 µM carnosic acid. Values indicate mean ± SEM. Statistics conducted using a paired *t*-test.

Kv7.2/3	$V_{0.5}$ Normalized tail current (mV)
Control	-29.25 ± 0.86 (*n* = 10)
100 μM Carnosic acid	-35.19 ± 0.88 (*n* = 10; *p*<0.0001)

Supplementary Table 21: Kv7.2/3 in the absence of or presence of 100 μM carnosic acid. Values indicate mean ± SEM. Statistics conducted using a paired *t*-test.

Kv7.3*	$V_{0.5}$ Normalized tail current (mV)
Control	-52.17 ± 1.54 (*n* = 7)
5 μM Retigabine	-93.94 ± 2.99 (*n* = 7; *p*<0.0001)
5 μM Retigabine<br>5 μM Carnosic acid	-115.03 ± 1.06 (*n* = 7; *p*<0.0001)

Supplementary Table 22: Kv7.3* in the absence of or presence of 5 μM retigabine and 5 μM retigabine + 5 μM carnosic acid. Values indicate mean ± SEM. Statistics conducted using a paired *t*-test.

Kv7.3/5	$V_{0.5}$ Normalized tail current (mV)
Control	-34.66 ± 2.63 (*n* = 7)
5 μM Carnosic acid	-51.55 ± 3.28 (*n* = 7; *p*<0.0001)

Supplementary Table 23: Kv7.3/5 in the absence of or presence of 5 μM carnosic acid. Values indicate mean ± SEM. Statistics conducted using a paired *t*-test.

Kv7.3/5	$V_{0.5}$ Normalized tail current (mV)
Control	-33.68 ± 1.73 (*n* = 7)
5 μM Retigabine	-50.04 ± 0.93 (*n* = 7; *p*<0.0001)
5 μM Retigabine<br>5 μM Carnosic acid	-59.17 ± 1.61 (*n* = 7; *p*<0.0001)

Supplementary Table 24: Kv7.3/5 in the absence of or presence of 5 μM retigabine and 5 μM retigabine + 5 μM carnosic acid. Values indicate mean ± SEM. Statistics conducted using a paired *t*-test.

**Kv7.2/3**	**$V_{0.5}$ Normalized tail current (mV)**
Control	-45.69 ± 1.27<br>(*n* = 8)
5 μM Retigabine	-69.72 ± 1.78<br>(*n* = 8; *p*<0.0001)
5 μM Retigabine<br>100 μM Carnosic acid	-99.09 ± 2.94<br>(*n* = 8; *p*<0.0001)

Supplementary Table 25: Kv7.2/3 in the absence of or presence of 5 μM retigabine and 5 μM retigabine + 100 μM carnosic acid. Values indicate mean ± SEM. Statistics conducted using a paired *t*-test.

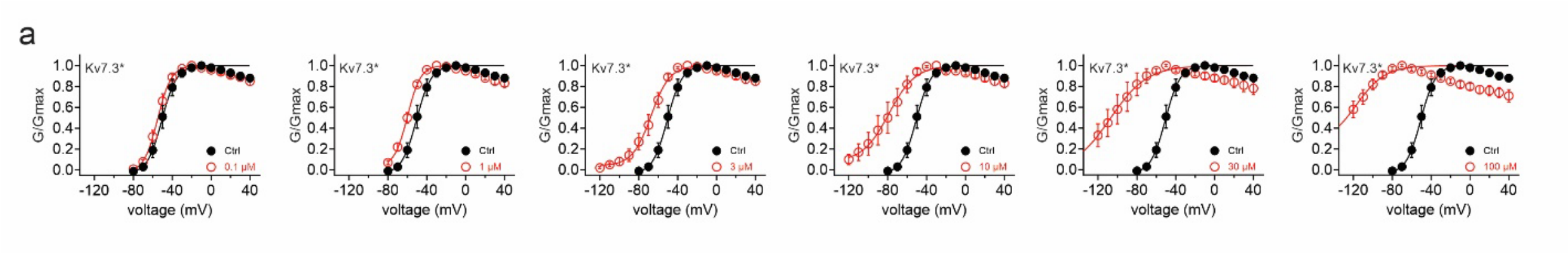

Supplementary Figure S1. Normalized conductance-voltage curves generated for Kv7.3* carnosic acid dose response studies.

A. Mean normalized tail current (G/Gmax) for Kv7.3* channels expressed in oocytes in the absence (Control; *black*) or presence of carnosic acid (0.1-100 µM); *n* = 5 per group.

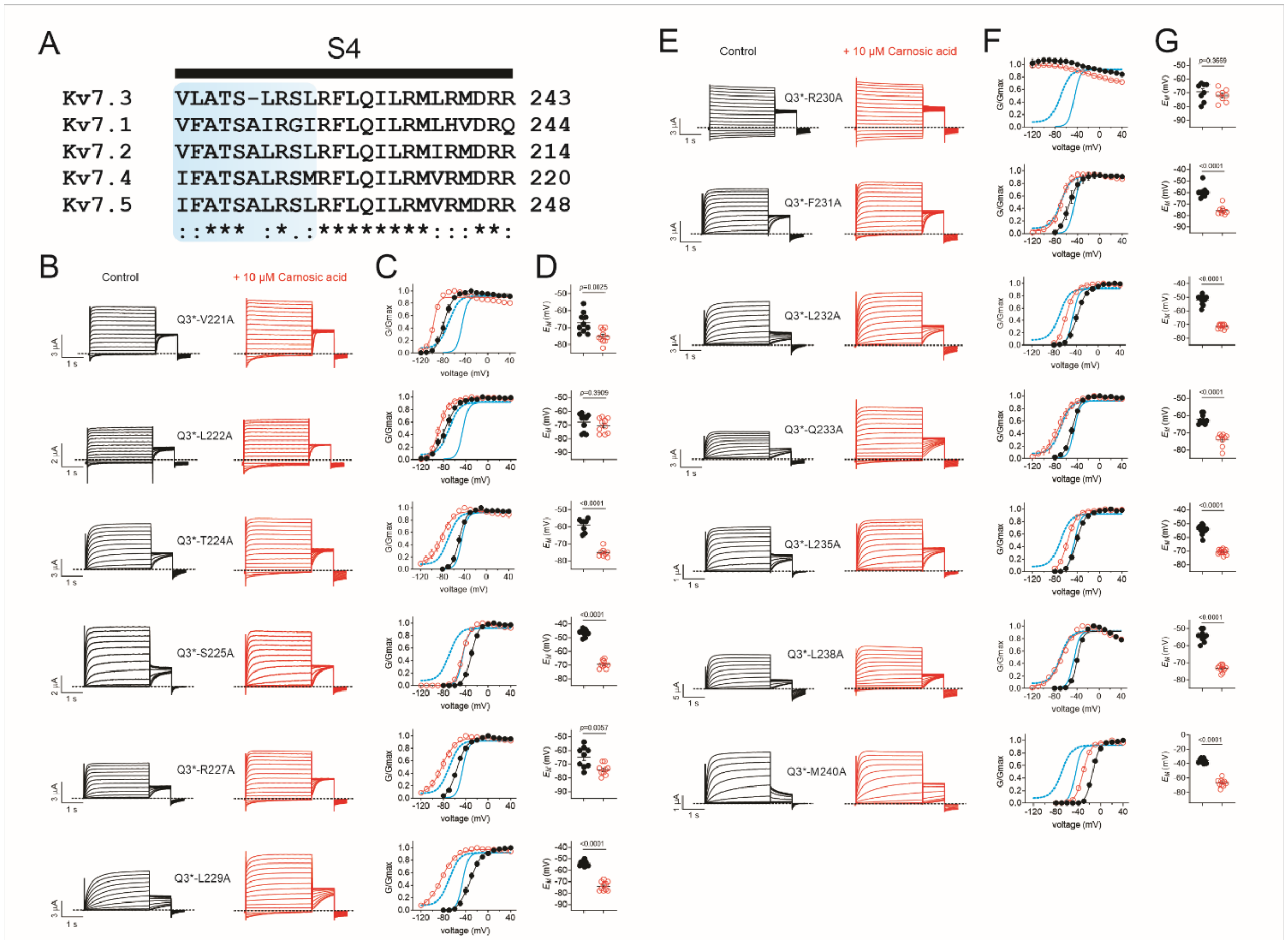

Supplementary Figure S2. Alanine scanning mutagenesis of Kv7.3 S4 domain identifies key residues for carnosic acid sensitivity.

A. Sequence alignment of human Kv7 S4 segments with the extracellular interface highlighted (blue).
B. Mean traces for Kv7.3* mutant V221A to L229A homomeric channels expressed in oocytes in the absence (Control; *black*) or presence of carnosic acid (10 µM) (*red*) ; *n* = 7-18 per group. Scale bars lower left for each trace.
C. Mean normalized tail current (G/Gmax) for traces as in **A**; *n* = 7-18 per group. Wild-type Kv7.3* fit lines overlaid for comparison, in the absence (solid purple) or presence (dashed purple) of carnosic acid (10 µM).
D. Mean unclamped oocyte membrane potential for traces as in **A**; *n* = 7-18 per group. Statistics conducted using a paired *t*-test.
E. Mean traces for Kv7.3* mutant R230A to M240A homomeric channels expressed in oocytes in the absence (Control; *black*) or presence of carnosic acid (10 µM) (*red*) ; *n* = 7-18 per group. Scale bars lower left for each trace.
F. Mean normalized tail current (G/Gmax) for traces as in **D**; *n* = 7-18 per group. Wild-type Kv7.3* fit lines overlaid for comparison, in the absence (solid purple) or presence (dashed purple) of carnosic acid (10 µM).
G. Mean unclamped oocyte membrane potential for traces as in **D**; *n* = 7-18 per group. Statistics conducted using a paired *t*-test.

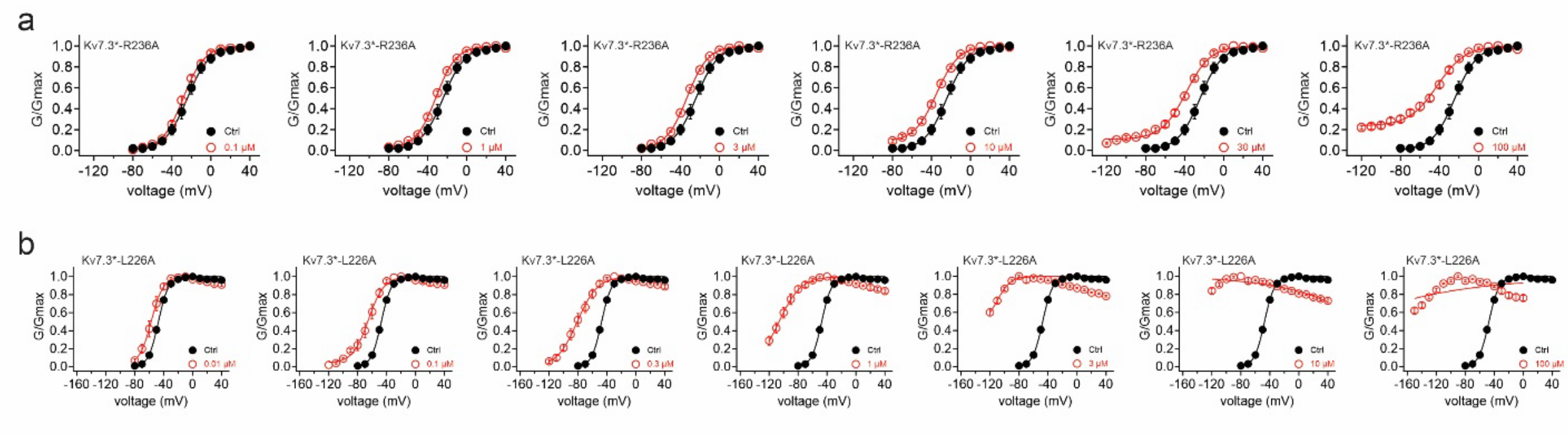

Supplementary Figure S3. Normalized conductance-voltage curves generated for Kv7.3*-R236A and Kv7.3*-L226A carnosic acid dose response studies.

A. Mean normalized tail current (G/Gmax) for Kv7.3*-R236A channels expressed in oocytes in the absence (Control; *black*) or presence of carnosic acid (0.1-100 µM); *n* = 6 per group.
B. Mean normalized tail current (G/Gmax) for Kv7.3*-L226A channels expressed in oocytes in the absence (Control; *black*) or presence of carnosic acid (0.01-100 µM); *n* = 7 per group.

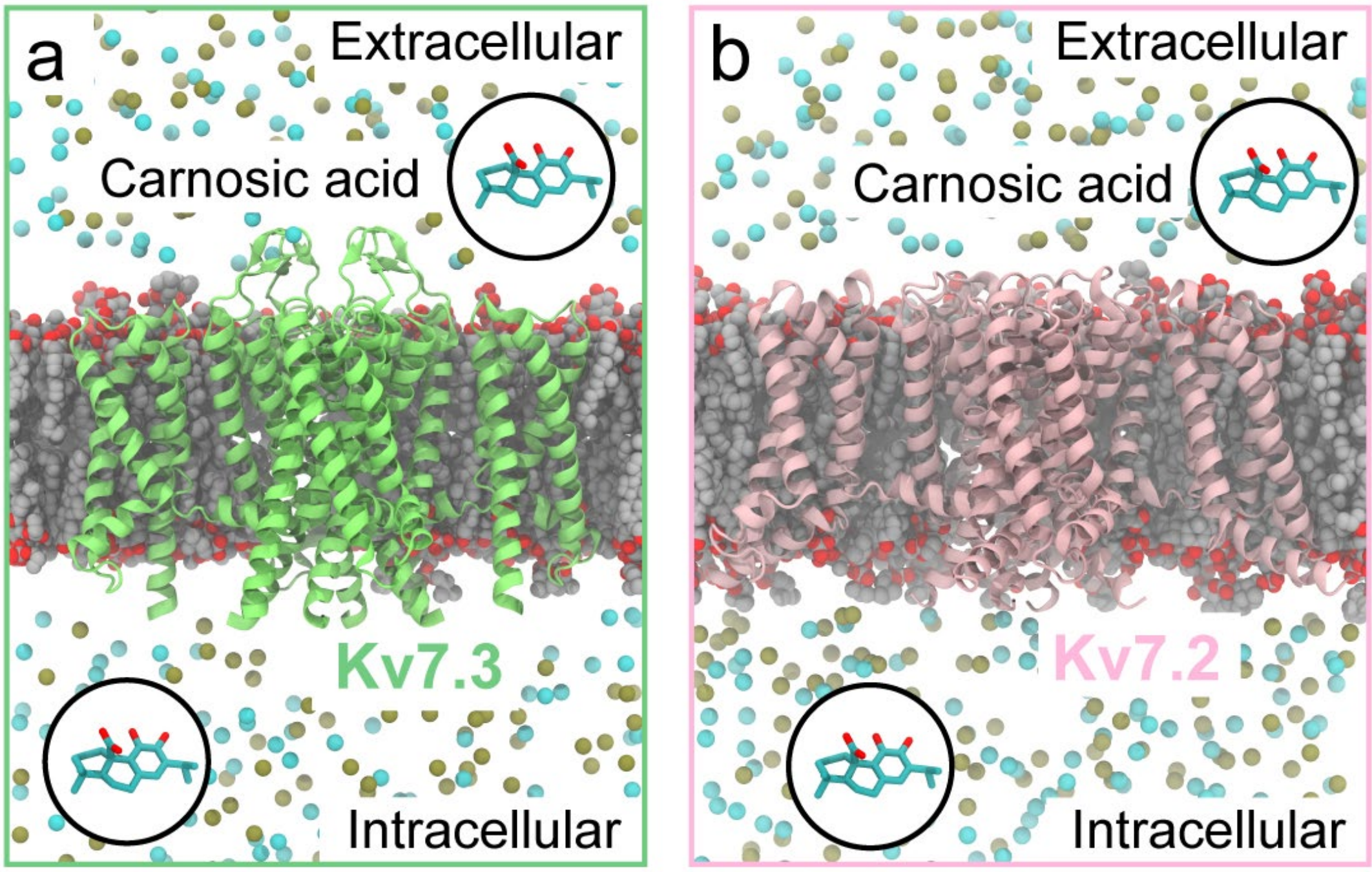

Supplementary Figure S4. All-atom molecular dynamics simulations of Kv7.3 and Kv7.2.

A. Atomic model of the Kv7.3 channel embedded in a heterogeneous lipid bilayer with lipid composition similar to that of a neuronal membrane. The Kv7.3 model was built using AlphaFold2 and aligned to a cryo-EM structure of Kv7.2 (PDB: 7CR0). Six molecules of carnosic acid were added in bulk solvent, along with 150 mM KCl. Two of the six carnosic acid molecules highlighted for clarity. Potassium and chloride ions are shown in ochre and cyan.

B. Atomic model of the Kv7.2 channel derived from a cryo-EM structure (PDB: 7CR0), with missing residues modeled using AlphaFold2. Membrane lipid composition, ion concentrations, and the number of carnosic acid molecules are identical to those described in A.

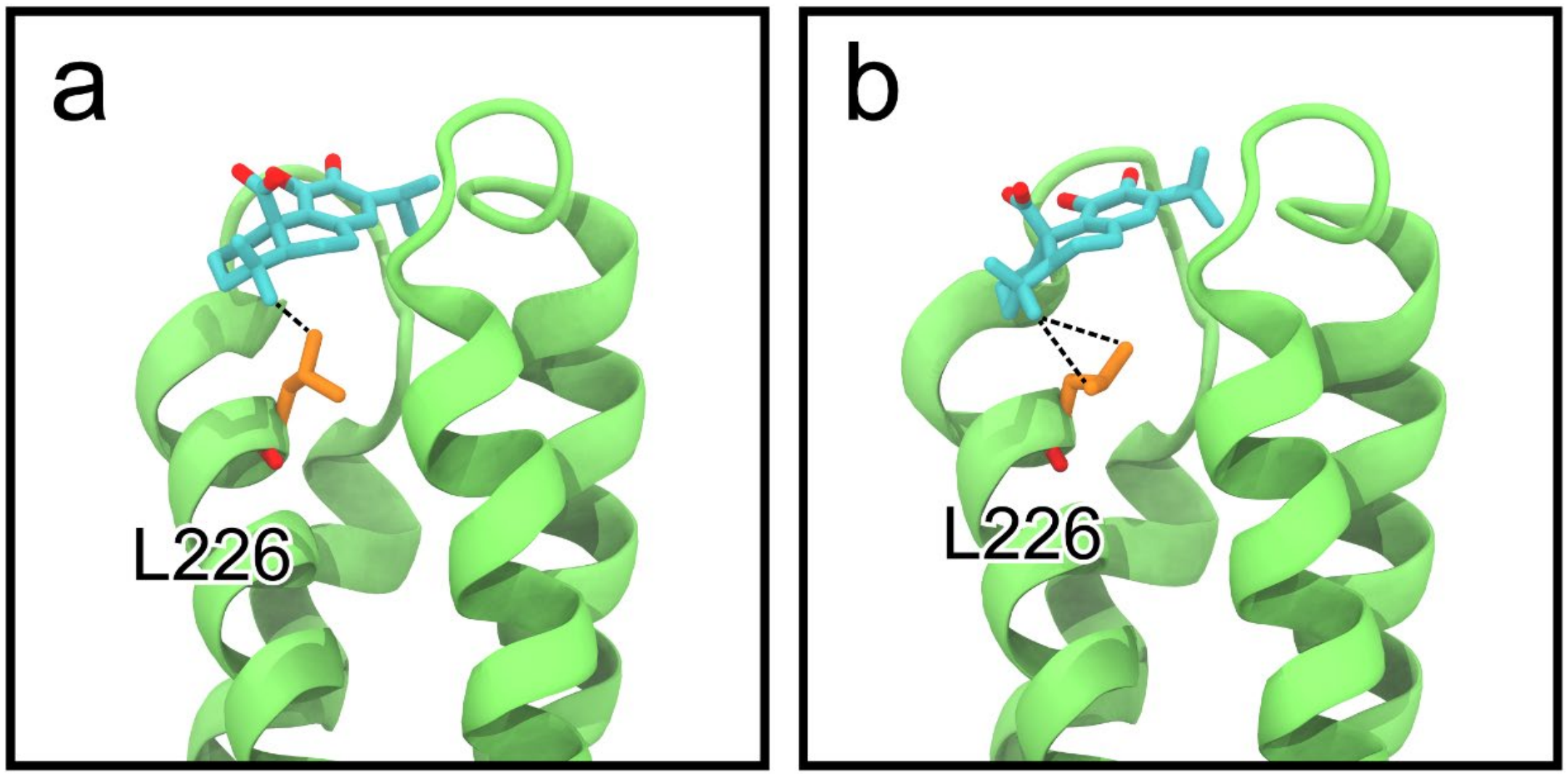

Supplementary Figure S5. The L226 side chain rotation acts as a hydrophobic latch that stabilizes carnosic acid binding.

A. L226 is rendered in a metastable configuration when CA enters the VSD binding pocket.
B. L226 is shown in a stable configuration once CA binds. Rotation of the L226 side chain stabilizes the CA pose and enables the ligand to insert more deeply into the binding pocket.

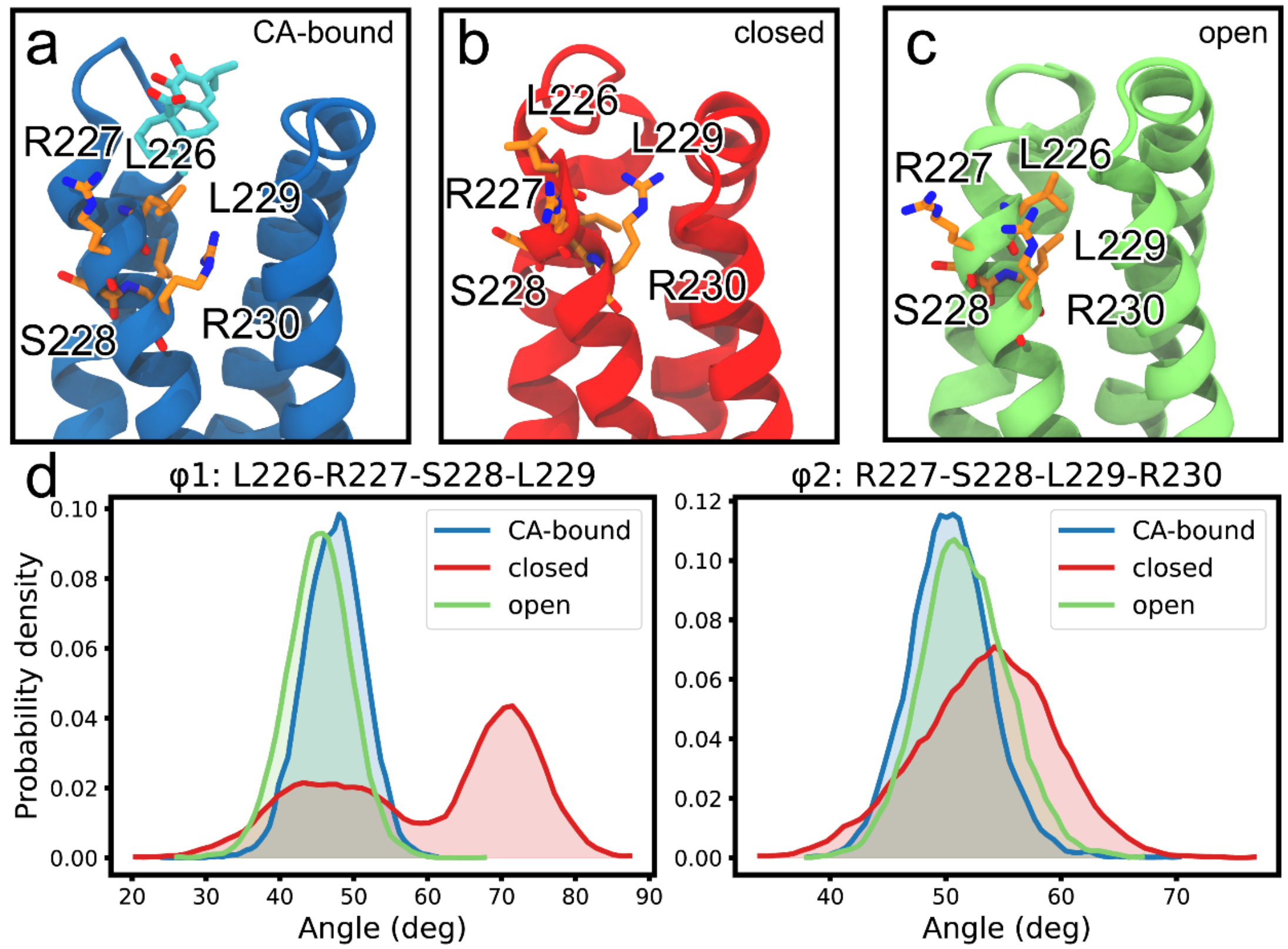

Supplementary Figure S6. S228 modulates the side chain orientations of L226, R227, and R230 in the CA binding pocket of Kv7.3.

A. Zoomed-in view of the Kv7.3 VSD revealing contacts between CA and L226, R227, and R230. S228 does not contact the ligand.
B. Zoomed-in view of the Kv7.3 VSD of residues L226, R227, S228, and R230 in the closed Kv7.3 conformation.
C. Zoomed-in view of the Kv7.3 VSD showing residues L226, R227, S228, and R230 in the open Kv7.3 conformation.
D. The distribution of dihedral angles involving S228. φ_1 denotes the angle defined by the Cα atoms of L226, R227, S228, and L229 and φ_2 denotes the angle formed by the Cα atoms of R227, S228, L229, and R230.

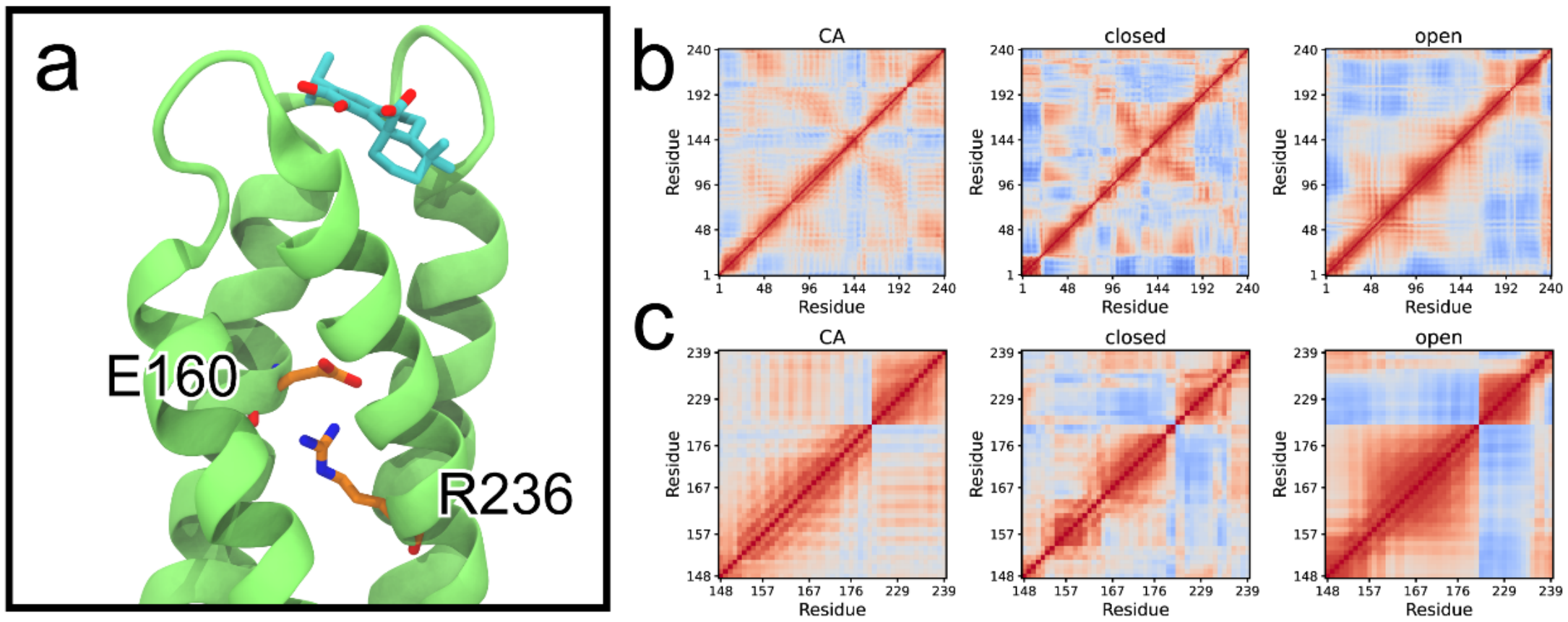

Supplementary Figure S7. R236 forms a salt bridge with E160 to couple the S2 and S4 helices of the VSD.

A. Zoomed-in view of the VSD showing residues R236 and E160, which do not form contacts with CA but instead form a salt bridge between them that correlates the motion of helix S2 with that of helix S4.
B. Dynamic cross-correlation matrices (DCCM) of a trajectory with CA bound to the VSD of Kv7.3 (left) and trajectories without CA, with the Kv7.3 channel in the closed state (center) and with the Kv7.3 channel in the open state (right). Color bar goes from negative correlation values (blue) to positive correlation value (red).
C. DCCM as in (B), restricted to residues on S2 (residues 148–181) and S4 (residues 224–240).

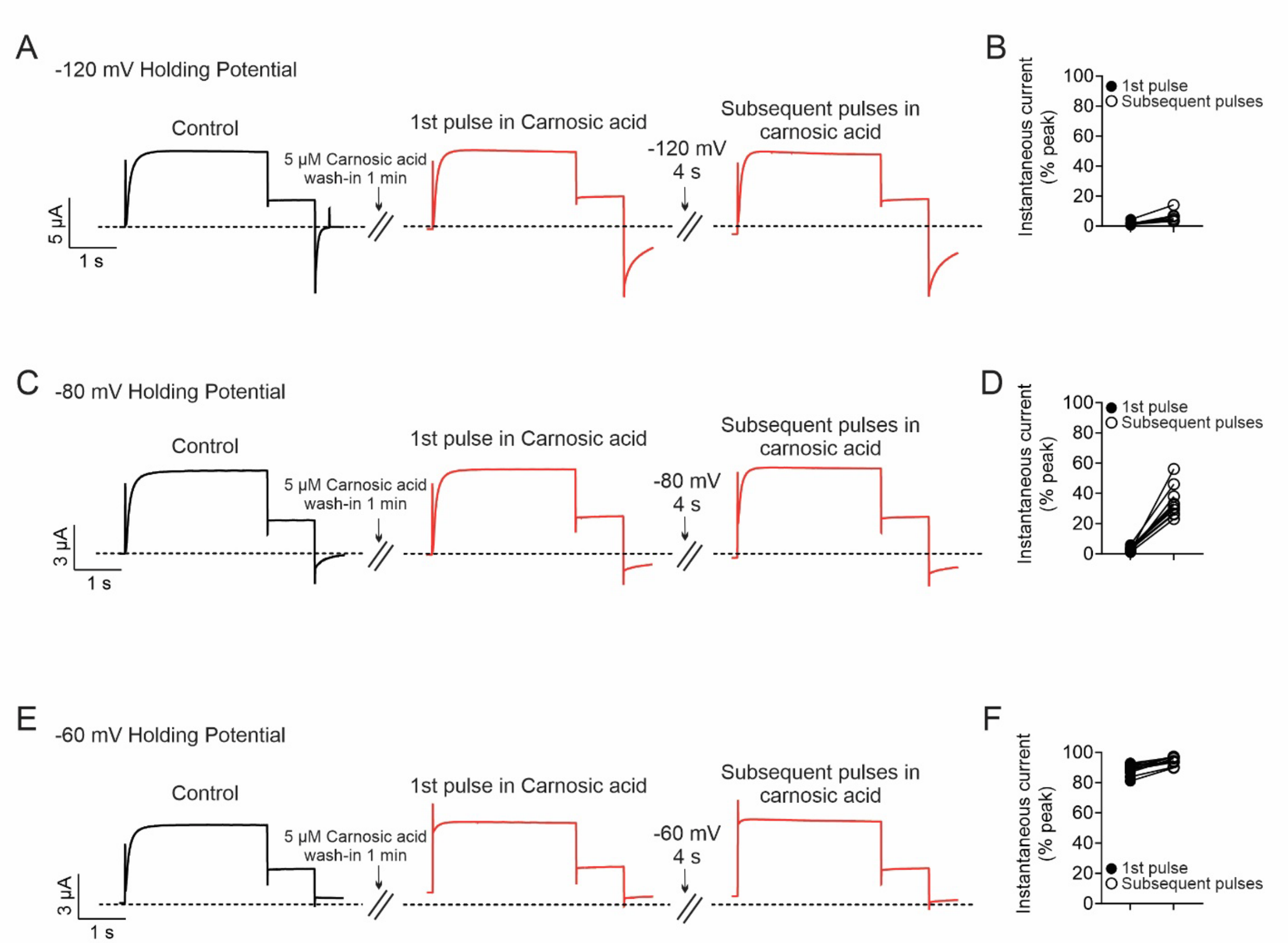

Supplementary Figure S8. State-dependent activity of carnosic acid at Kv7.3*.

A. Exemplar recordings of Kv7.3* channels expressed in *Xenopus* oocytes in control bath solution (black) held at -120 mV and depolarized to +40 mV. Cells continued to be held at -120 mV for 1 min during extracellular application of 5 μM carnosic acid. Cells are then depolarized to +40 mV (first pulse in carnosic acid), with repeated depolarizations every 4 s (subsequent pulses in carnosic acid).
B. Instantaneous current in the first pulse and subsequent pulses as a percentage of the peak current calculated from **A**; *n* = 8 per group.
C. Exemplar recordings of Kv7.3* channels expressed in *Xenopus* oocytes in control bath solution (black) from a holding potential of -80 mV.
D. Instantaneous current in the first pulse and subsequent pulses as a percentage of the peak current calculated from **C**; *n* = 10 per group.
E. Exemplar recordings of Kv7.3* channels expressed in *Xenopus* oocytes in control bath solution (black) from a holding potential of -60 mV.
F. Instantaneous current in the first pulse and subsequent pulses as a percentage of the peak current calculated from **E**; *n* = 10 per group.

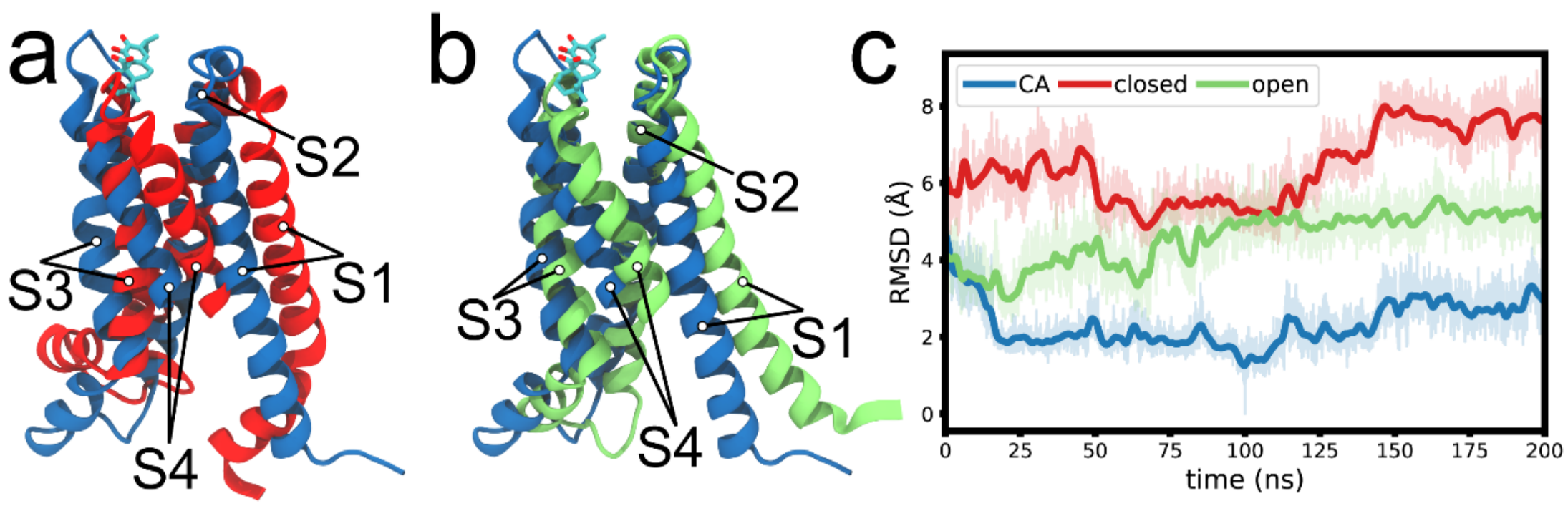

Supplementary Figure S9. Structural alignment and conformational stability of the Kv7.3 VSD with CA and in the open and closed states.

A. Structural alignment of the simulated CA bound Kv7.3 VSD (blue) and the closed apo conformation (red). The S2 helix is used as the reference of the alignment.
B. Structural alignment of the simulated CA bound Kv7.3 VSD (blue) and the open apo conformation (green). The S2 helix is used as the reference of the alignment.
C. Root-mean-square deviation (RMSD) of the VSD during MD simulations in which CA binds to the VSD (blue), the closed state of Kv7.3 (green), and the open state of Kv7.3 (red). The CA-bound VSD display conformational dynamics that are more structurally more similar to the open state than the closed state.

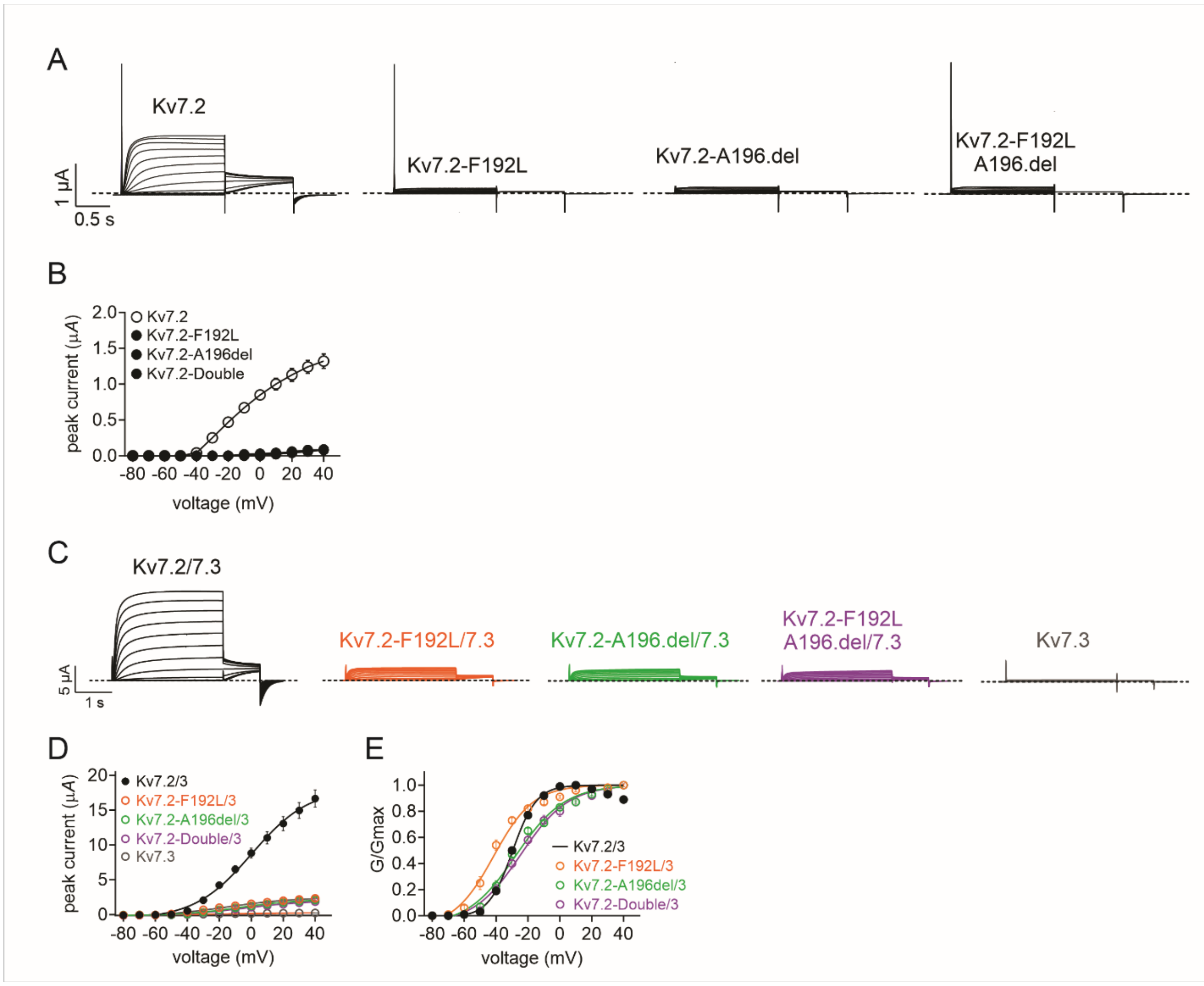

Supplementary Figure S10. Baseline characterization of homomeric Kv7.2 mutant channels and heteromeric Kv7.2 mutant/7.3 channels.

A. Mean traces for Kv7.2 wild type (*n* = 6), Kv7.2-F192L (*n* = 7), Kv7.2-A196del (*n* = 7), and Kv7.2-F192L, A196del (*n* = 7) homomeric channels expressed in oocytes. Scale bars lower left for each trace.
B. Mean peak prepulse current for traces as in **A**; *n* = 6-7 per group.
C. Mean traces for Kv7.2/3 wild type (*n* = 8), Kv7.2-F192L/7.3 (*n* = 8), Kv7.2-A196del/7.3 (*n* = 8), and Kv7.2-F192L, A196del/7.3 (*n* = 9) heteromeric channels and homomeric Kv7.3 wild type channels (*n* = 10) expressed in oocytes. Scale bars lower left for each trace.
D. Mean peak prepulse current for traces as in **C**; *n* = 8-10 per group.
E. Mean normalized tail current (G/Gmax) for traces as in **C**; *n* = 8-10 per group.

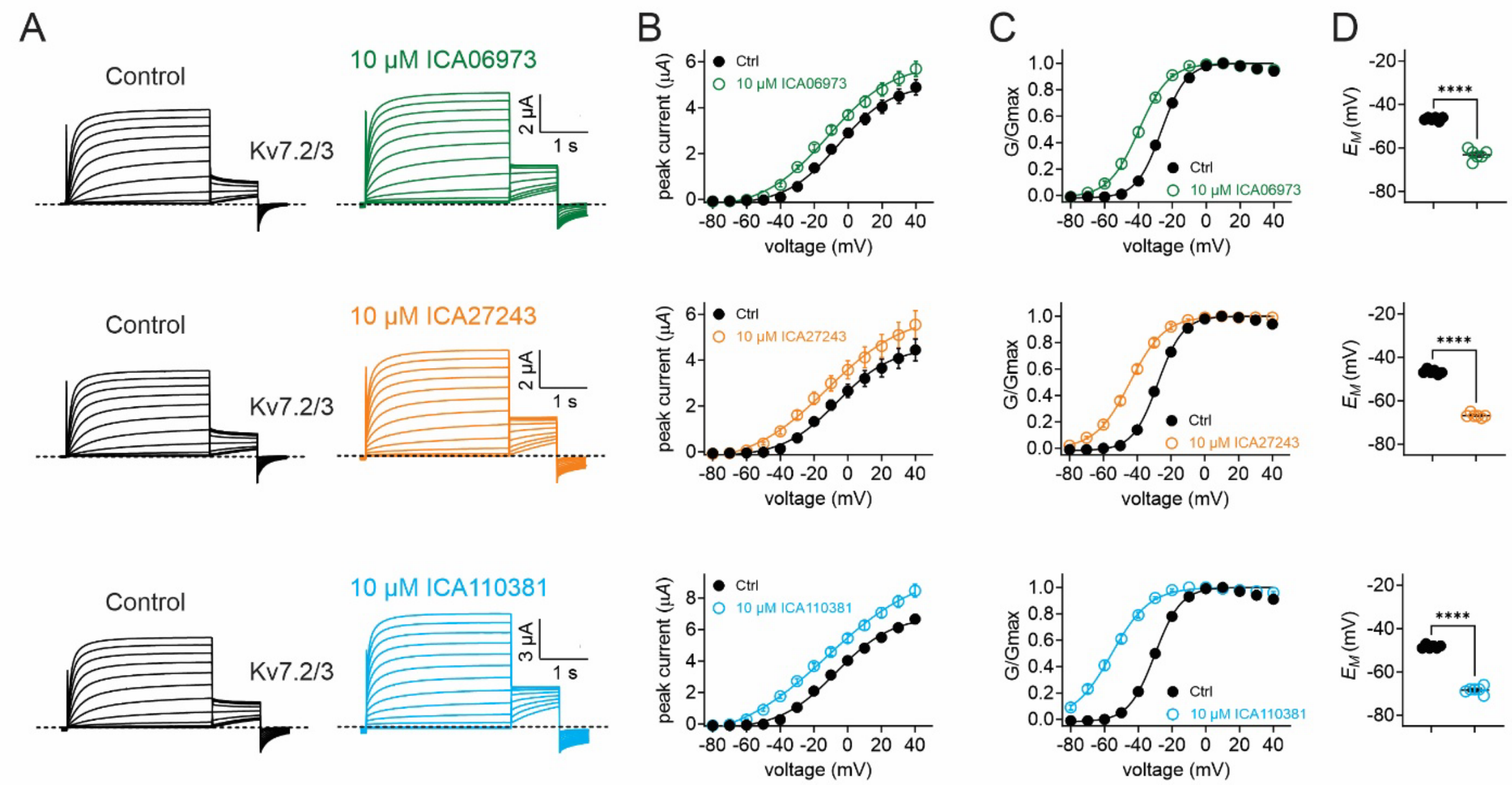

Supplementary Figure S11. Characterization of the effects of ICA compounds on wild type Kv7.2/3 channels.

A. Mean traces for Kv7.2/3 channels expressed in oocytes in the absence (Control; *black*) or presence of 10 µM ICA06973 (*green*), ICA27243 (*orange*), and ICA110381 (*blue*); *n* = 6 per group. Scale bars upper right for each trace.
B. Mean peak prepulse current for traces as in **A**; *n* = 6 per group.
C. Mean normalized tail current (G/Gmax) for traces as in **A**; *n* = 6 per group.
D. Mean unclamped oocyte membrane potential for traces as in **A**; *n* = 6 per group. Statistics conducted using a paired *t*-test.

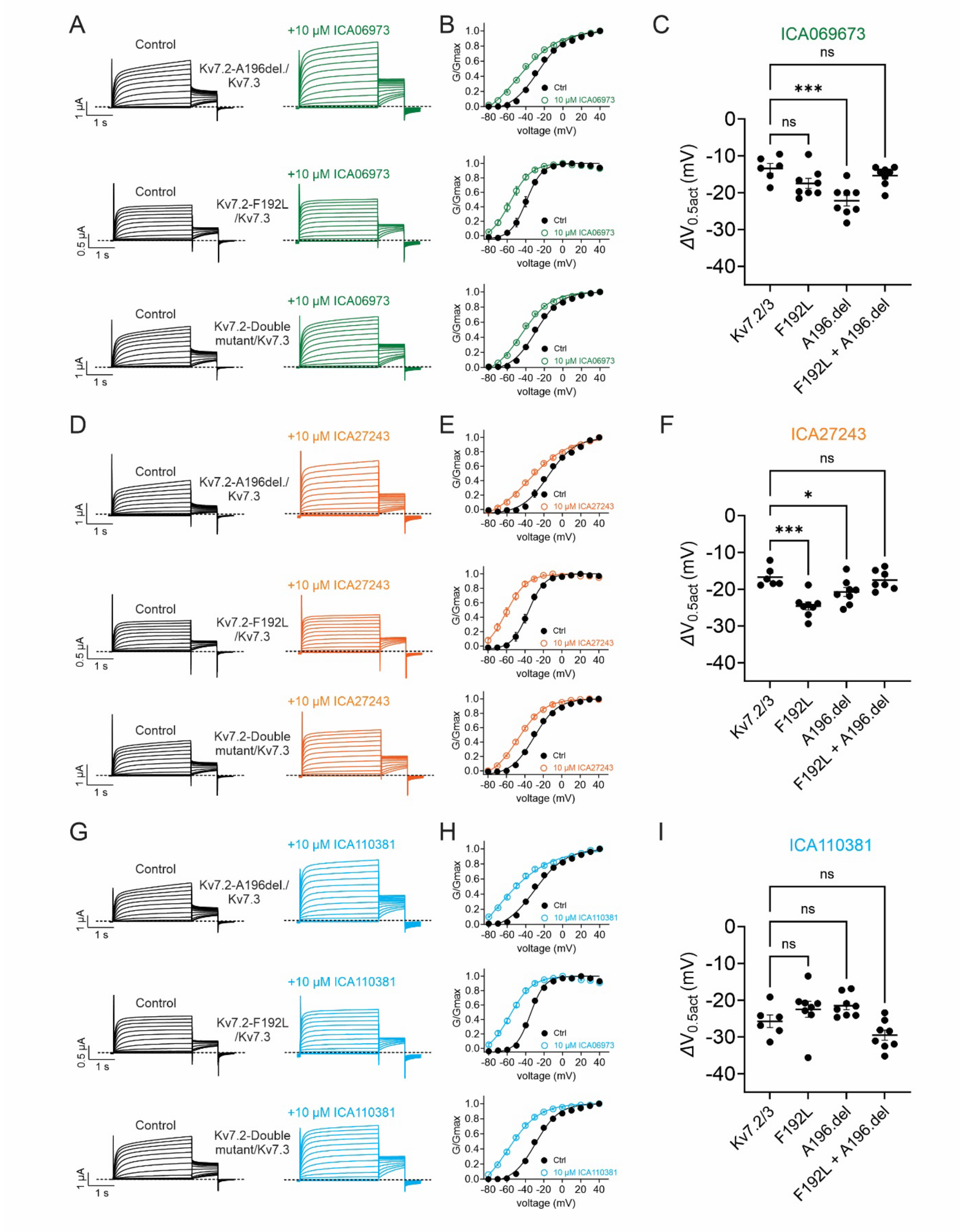

Supplementary Figure S12. Characterization of the effects of ICA compounds at heteromeric Kv7.2 mutant/7.3 channel complexes.

A. Mean traces for Kv7.2-A196del/7.3, Kv7.2-F192L/7.3, and Kv7.2-F192L, A196del/7.3 heteromeric channels expressed in oocytes in the absence (black) or presence (green) of 10 μM ICA069673; *n* = 8 per group. Scale bars lower left for each trace.
B. Mean normalized tail current (G/Gmax) for traces as in **A**; *n* = 8 per group.
C. Summary of the $\Delta V_{0.5act}$ shift for 10 μM ICA069673 at Kv7.2-A196del, Kv7.2-F192L, and Kv7.2-F192L, A196del heteromeric channels versus wild type Kv7.2/3; *n* = 6-8 per group.
D. Mean traces for Kv7.2-A196del/7.3, Kv7.2-F192L/7.3, and Kv7.2-F192L, A196del/7.3 heteromeric channels expressed in oocytes in the absence (black) or presence (orange) of 10 μM ICA27243; *n* = 7-8 per group. Scale bars lower left for each trace.
E. Mean normalized tail current (G/Gmax) for traces as in **D**; *n* = 7-8 per group.
F. Summary of the $\Delta V_{0.5act}$ shift for 10 μM ICA27243 at Kv7.2-A196del, Kv7.2-F192L, and Kv7.2-F192L, A196del heteromeric channels versus wild type Kv7.2/3; *n* = 6-8 per group.
G. Mean traces for Kv7.2-A196del/7.3, Kv7.2-F192L/7.3, and Kv7.2-F192L, A196del/7.3 heteromeric channels expressed in oocytes in the absence (black) or presence (cyan) of 10 μM ICA110381; *n* = 8 per group. Scale bars lower left for each trace.
H. Mean normalized tail current (G/Gmax) for traces as in **G**; *n* = 8 per group.
I. Summary of the $\Delta V_{0.5act}$ shift for 10 μM ICA110381 at Kv7.2-A196del, Kv7.2-F192L, and Kv7.2-F192L, A196del heteromeric channels versus wild type Kv7.2/3; *n* = 6-8 per group.

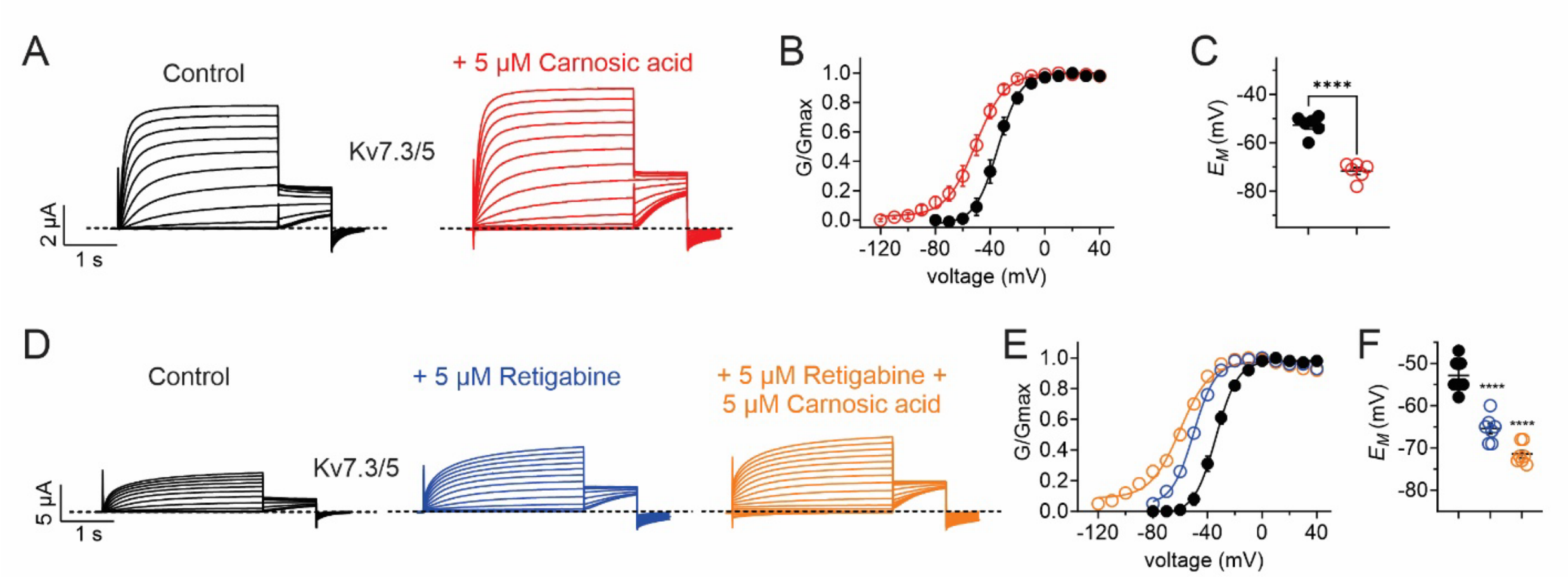

Supplementary Figure S13. Action of carnosic acid and retigabine on Kv7.3/5 heteromeric channels.

A. Mean traces for Kv7.3/5 expressed in oocytes in the absence (Control; *black*) or presence of 5 µM carnosic acid (*red*); *n* = 7 per group. Scale bars lower left for each trace.
B. Mean normalized tail current (G/Gmax) for traces as in **A**; *n* = 7 per group.
C. Mean unclamped oocyte membrane potential as in **A**; *n* = 7 per group. Statistics conducted using a paired *t*-test.
D. Mean traces for Kv7.3/5 expressed in oocytes in the absence (Control; *black*) or presence of 5 µM retigabine (*blue*; n = 7) or 5 µM carnosic acid and 5 µM retigabine (*orange*; n = 7). Scale bars lower left for each trace.
E. Mean normalized tail current (G/Gmax) for traces as in **D**; *n* = 7 per group.
F. Mean unclamped oocyte membrane potential as in **D**; *n* = 7 per group. Statistics conducted using a paired *t*-test.

References

1. Grosdidier, A., Zoete, V. & Michielin, O. Fast docking using the CHARMM force field with EADock DSS. *Journal of Computational Chemistry* **32**, 2149–2159 (2011).
2. The PyMOL Molecular Graphics System, Version 3.0 Schrödinger, LLC.
3. Humphrey, W., Dalke, A. & Schulten, K. VMD: Visual molecular dynamics. *Journal of Molecular Graphics* **14**, 33–38 (1996).
4. Ingólfsson, H. I. *et al.* Computational Lipidomics of the Neuronal Plasma Membrane. *Biophysical Journal* **113**, 2271–2280 (2017).
5. Jo, S., Lim, J. B., Klauda, J. B. & Im, W. CHARMM-GUI Membrane Builder for Mixed Bilayers and Its Application to Yeast Membranes. *Biophysical Journal* **97**, 50–58 (2009).
6. Wu, E. L. *et al.* CHARMM-GUI Membrane Builder toward realistic biological membrane simulations. *Journal of Computational Chemistry* **35**, 1997–2004 (2014).
7. Jo, S., Kim, T. & Im, W. Automated Builder and Database of Protein/Membrane Complexes for Molecular Dynamics Simulations. *PLOS ONE* **2**, e880 (2007).
8. Huang, J. *et al.* CHARMM36m: an improved force field for folded and intrinsically disordered proteins. *Nat Methods* **14**, 71–73 (2017).
9. MacKerell, A. D. Jr. *et al.* All-Atom Empirical Potential for Molecular Modeling and Dynamics Studies of Proteins. *J. Phys. Chem. B* **102**, 3586–3616 (1998).
10. Jorgensen, W. L., Chandrasekhar, J., Madura, J. D., Impey, R. W. & Klein, M. L. Comparison of simple potential functions for simulating liquid water. *J. Chem. Phys.* **79**, 926–935 (1983).
11. Vanommeslaeghe, K. *et al.* CHARMM general force field: A force field for drug-like molecules compatible with the CHARMM all-atom additive biological force fields. *Journal of Computational Chemistry* **31**, 671–690 (2010).
12. Andersen, H. C. Rattle: A "velocity" version of the shake algorithm for molecular dynamics calculations. *Journal of Computational Physics* **52**, 24–34 (1983).
13. Tuckerman, M., Berne, B. J. & Martyna, G. J. Reversible multiple time scale molecular dynamics. *J. Chem. Phys.* **97**, 1990–2001 (1992).
14. Essmann, U. *et al.* A smooth particle mesh Ewald method. *J. Chem. Phys.* **103**, 8577–8593 (1995).
15. Phillips, J. C. *et al.* Scalable molecular dynamics with NAMD. *Journal of Computational Chemistry* **26**, 1781–1802 (2005).
16. K. Kasahara, I. Fukuda, H. Nakamura, A novel approach of dynamic cross correlation analysis on molecular dynamics simulations and its application to ETS1 Dimer–DNA Complex. *PLoS ONE* **9**, e112419 (2014).
